# Artificial Metalloenzymes based on TetR Proteins and Cu(II) for Enantioselective Friedel‐Crafts Alkylation Reactions

**DOI:** 10.1002/cctc.202000245

**Published:** 2020-04-29

**Authors:** Cora Gutiérrez de Souza, Manuela Bersellini, Gerard Roelfes

**Affiliations:** ^1^ Stratingh Institute for Chemistry University of Groningen Nijenborgh 49747 AG Groningen The Netherlands

**Keywords:** artificial metalloenzymes, biocatalysis, Friedel-Crafts alkylation, Cu(II) ion, TetR proteins

## Abstract

The supramolecular approach is among the most convenient methodologies for creating artificial metalloenzymes (ArMs). Usually this approach involves the binding of a transition metal ion complex to a biomolecular scaffold *via* its ligand, which also modulates the catalytic properties of the metal ion. Herein, we report ArMs based on the proteins CgmR, RamR and QacR from the TetR family of multidrug resistance regulators (MDRs) and Cu^2+^ ions, assembled without the need of a ligand. These ArMs catalyze the enantioselective vinylogous Friedel‐Crafts alkylation reaction with up to 75 % ee. Competition experiments with ethidium and rhodamine 6G confirm that the reactions occur in the chiral environment of the hydrophobic pocket. It is proposed that the Cu^2+^‐substrate complex is bound via a combination of electrostatic and π‐stacking interactions provided by the second coordination sphere. This approach constitutes a fast and straightforward way to assemble metalloenzymes and may facilitate future optimization of the protein scaffolds via mutagenesis or directed evolution approaches.

## Graphical abstract

**Biocatalysis**: A new class of artificial metalloenzymes based on TetR proteins and Cu(II) ions, without the need for an external additional ligand for the metal, for the catalysis of enantioselective Friedel‐Crafts alkylation of indoles.

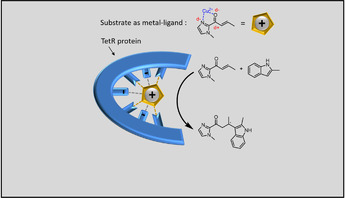

The field of artificial metalloenzymes (ArMs) has the potential to expand dramatically the possibilities of enzyme engineering towards achieving biocatalysis of reactions that have no equivalent in nature.^1^, ^2^, ^3^ ArMs aim to combine the high efficiency of natural enzymes, with the broad reaction scope characteristic of homogeneous transition metal catalysts. ArMs consist of a biological scaffold (DNA or protein) in which a catalytically active transition metal complex is embedded. The bioscaffold provides the second coordination sphere interactions that are envisioned to contribute to the rate acceleration of the reaction and a chiral environment that allows catalytic transformations to proceed in an enantioselective manner. The metal complex is responsible for the catalytic activity. A key role is held by the ligand, which modulates the electronic properties of the metal ion and, hence, attenuates the reactivity. Additionally, the ligand can be used to anchor the metal complex inside the biomolecular scaffold *via* a tethered “Trojan horse” moiety, as applied successfully in the (strept‐)avidin/biotin based systems, or directly by making use of supramolecular interactions between the ligand and the protein scaffold.^4^, ^5^, ^6^, ^7^, ^8^, ^9^, ^10^, ^11^ The supramolecular approach is attractive because of its simplicity, since the system self‐assembles upon mixing the protein and the metal complex. This approach does not require covalent modifications of the scaffold and thus facilitates ArM optimization. Nevertheless, it is limited in the scope of proteins that can be used as scaffold, as strong and specific interactions between the protein and the cofactor are generally required.^11^, ^12^, ^13^, ^14^

In our group, we have applied the supramolecular approach to develop ArMs based on the protein LmrR (Lactococcal Multidrug Resistance Regulator) from the PadR family of Multidrug Resistance Regulators (MDRs).^15^, ^16^, ^17^ LmrR is a homodimeric protein with a spacious hydrophobic pocket at the dimer interface that captures aromatic compounds by π‐stacking interactions in between the indole moieties of tryptophans W96 and W96’, located in the middle of the pore (Figure 1a). The affinity of these two tryptophans for binding planar metal complexes, *e. g*. the Cu^2+^ complex of 1,10‐phenanthroline (Cu(phen)), was exploited to create LmrR‐based ArMs for the enantioselective Friedel‐Crafts alkylation of α‐β unsaturated acyl imidazole with indole.^18^

**Figure 1:**
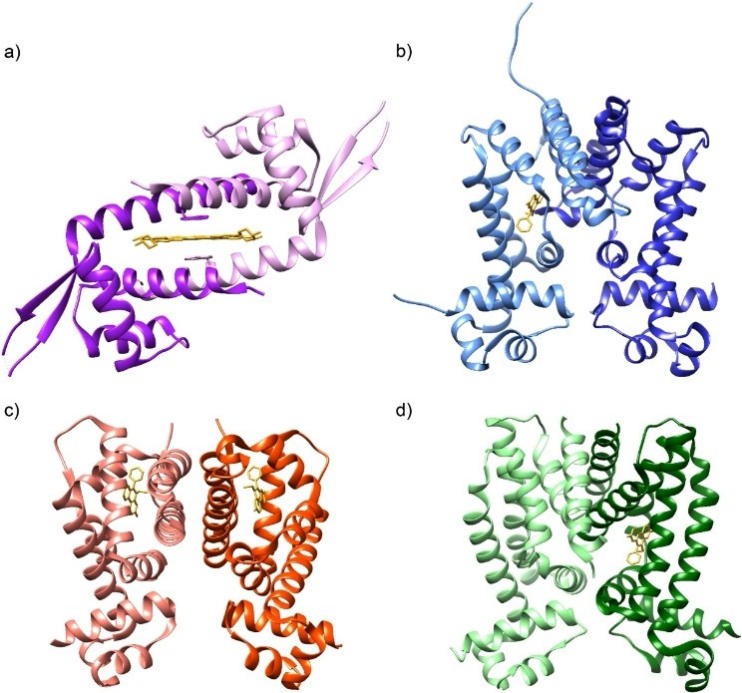
Cartoon representations of a) LmrR with Hoechst 33342 (PDB: 3F8C). b) CgmR with ethidium bromide (PDB: 2ZOZ). c) RamR with ethidium bromide (PDB: 3VVY). d) QacR with ethidium bromide (PDB: 1JTY).

In the current study, we aimed to expand the array of bioscaffolds to other MDRs, in particular proteins belonging to the TetR family, which are well‐studied bacterial transcriptional repressors.^19^ These are homodimeric proteins of ∼20 KDa per monomer that, unlike LmrR, have one binding pocket per monomer. We hypothesized that differences in the structures of the hydrophobic pockets could give rise to new ArMs with reactivities and selectivities complementary to that of LmrR. Here, we show that these novel ArMs are able to catalyze the enantioselective Friedel‐Crafts alkylation of α,β‐unsaturated imidazoles with indoles using Cu(NO_3_)_2_ alone, without the need of any ligand for the metal ion.

CgmR, RamR and QacR were selected as scaffolds for this study because crystal structures of these proteins with different hydrophobic and cationic drugs were available.^20^, ^21^, ^22^, ^23^, ^24^, ^25^, ^26^, ^27^ The ability of these proteins to bind aromatic and cationic molecules suggested their capability to capture hydrophobic metal complexes. Moreover, these protein scaffolds have previously proven their utility in artificial metalloenzyme design when combined with a metal‐binding unnatural amino acid.^28^

The ArMs were prepared by self‐assembly of CgmR, RamR and QacR with Cu(phen)(NO_3_)_2_ complex or Cu(NO_3_)_2_. In case of QacR, a variant containing C72A and C141S mutations was used.^28^ UV‐Vis studies suggested interaction between the proteins and these copper species (Figure S4.5), albeit no quantitative binding data could be obtained. The Friedel‐Crafts alkylation (F−C reaction) of 2‐methyl‐(1*H*)‐indole (**2 a**) with1‐(1‐methyl‐1*H*‐imidazole‐2‐yl)but‐2‐en‐1‐one (**1 a**) was selected as the model reaction. This reaction involves the conjugate addition of a heteroaromatic nucleophilic indole to an α,β‐unsaturated 2‐acyl‐(1‐methyl)imidazole substrate and it is typically catalyzed by a Lewis acid, *e. g*. Cu^2+^.^29^

Initial studies were performed using Cu(phen)(NO_3_)_2_ or Cu(NO_3_)_2_ (90 μM) with a slight excess of 1.3 equivalents of each protein (120 μM in protein dimer), 1 mM **1 a** and 1 mM **2 a** in 20 mM MOPS pH 7.0, containing 500 mM NaCl. The high salt concentrations were required to avoid precipitation of the proteins during the reaction. The reactions were incubated under continuous inversion at 4 °C for 72 hours. No product formation was observed in absence of Cu(NO_3_)_2_ or Cu(phen)(NO_3_)_2_. The reaction in presence of Cu(NO_3_)_2_ alone, without protein, resulted in formation of a racemic mixture of the Friedel‐Crafts product in 22 % yield, indicating that the Cu^2+^ ion is required for activation of the imidazole substrate. In addition, the proteins alone led to low yields (up to 11 %) and ee's. The yields are somewhat higher than observed without any catalyst, which might be due to non‐specific interactions of the substrates with the protein, resulting in a higher effective molarity.

The reaction catalyzed by Cu(phen)(NO_3_)_2_ gave a significantly higher yield compared to using Cu(NO_3_)_2_ as catalyst (Table 1, entries 2 and 3). Unexpectedly, the reactions performed with the MDR proteins in combination with Cu(phen)(NO_3_)_2_ resulted in lower yields (ranging from 21 to 36 % depending on the protein scaffold used) than with MDR/Cu(NO_3_)_2_ (52 to 78 % yield). The best results were obtained with QacR with 78 % yield and 34 % ee but similar enantioselectivities (Table 1, and Table S2). The inferior yields obtained with the TetR ArMs prepared with Cu(phen)(NO_3_)_2_ compared to those prepared from Cu(NO_3_)_2_, without ligand, was a surprising finding, since it is in marked contrast to what was previously reported for LmrR. For the latter, the presence of the ligand for the metal ion was required.^18^

**Table 1:** Vinylogous Friedel‐Crafts alkylation reactions catalyzed by MDR/Cu(II) or MDR/Cu(phen).

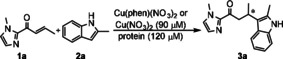			
Entry	Catalyst	Yield [%]^[a]^	ee [%]^[a]^
1	–	<5	–
2	Cu^2+^	22±7	–
3	Cu(phen)	43±2	–
4	RamR	5±6	8±6
5	Cu^2+^⊂RamR	57±9	29±3
6	Cu(phen)⊂RamR	21±13	34±7
7	CgmR	6±3	10±21
8	Cu^2+^⊂CgmR	52±12	13±3
9	Cu(phen)⊂CgmR	30±3	15±1
10	QacR	11±7	13±19
11	Cu^2+^⊂QacR	78±11	34±3
12	Cu(phen)⊂QacR	36±6	30±2

[a] Yields and ee's were determined by HPLC. Yields were calculated using 2‐phenylquinoline as internal standard. All the results listed correspond to the average of two independent experiments, each carried out in duplicate. Errors listed are standard deviations; [b] In all cases the (−) enantiomer was obtained in excess, as determined by comparison of the elution order in chiral HPLC to literature reports.^29^, ^30^

The enantioselectivities observed indicate that the catalytic reaction is occurring within the chiral environment of the proteins. Moreover, the higher yield obtained with Cu^2+^ + proteins compared to the reaction performed only with Cu^2+^ salt suggests that all the protein scaffolds also contribute to acceleration of the reaction.

To determine the substrate scope of these new ArMs, the F−C alkylation reaction was performed with the TetR/Cu^2+^ based ArMs with three α‐β‐substituted imidazoles (**1 a**–**c**) and a selection of substituted indoles (**2 a**–**f**, Table 2 and Table S1). The indole scope was tested on β‐methyl substituted imidazole substrate (**1 a**). 2‐methyl‐(1*H*)‐indole (**2 a**) was found to be the most reactive among the indoles, with yields and ee's of product **3 a** ranging from 52 to 78 % and from 13 to 34 %, respectively. 5‐methoxy‐(1*H*)‐indole (**2 d**) also showed good reactivity, giving rise to yields of **3 d** between 19 and 39 % and ee's between 9 and 37 %. Unsubstituted indole led to low yields of product **2 b**, whereas indoles harboring a phenyl moiety at position 2 and deactivating groups at position 5 of the indole ring, such as chlorine and bromine, were mostly unreactive (Table 2, entries 3, 5 and 6, and Table S1 entries 9–12 and 17–24).^18^ Only in case of Cu^2+^⊂QacR, a slightly increased yield of the reaction with 5‐chloro‐(1*H*)‐indole substrate (**2 e**) with a moderate enantioselectivity was found.

**Table 2:** Substrate scope of the vinylogous Friedel‐Crafts alkylation reactions.^[a]^

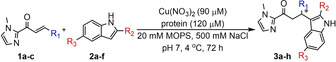					
Entry	Product	Y/ee [%]^[b]^ Cu^2+^ Cu^2+^⊂RamR Cu^2+^⊂CgmR Cu^2+^⊂QacR	Entry	Product	Y/ee [%]^[b]^ Cu^2+^ Cu^2+^⊂RamR Cu^2+^⊂CgmR Cu^2+^⊂QacR
1	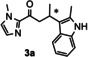		5	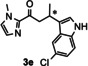	
	22±7/– 57±9/29±3 (−) 52±12/13±3 (−) 78±11/34±3 (−)			<5/n.d. <5/n.d. <5/n.d. 17±8/38±5 (+)	
2	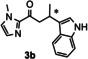		6	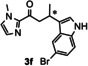	
	7±8/– 5/n.d. 7±3/27±3 (+)−*R* 27±12/26±2 (+)−*R*			<5/n.d. <5/n.d. <5/n.d. <5/n.d.	
3	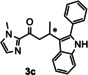		7	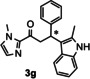	
	<5/n.d. <5/n.d. <5/n.d. <5/n.d.			54±9/– <5/n.d. 18±4/6±2 59±7/75±4	
4	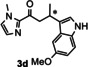		8	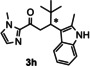	
	10±6/– 19±3/35±3 (+) 31±14/37±1 (+) 39±10/9±4 (+)			<5/n.d. <5/n.d. <5/n.d. <5/n.d.	

[a] Typical conditions: 90 μM Cu(NO_3_)_2_ (9 mol%) loading with 1.3 equivalents of protein (120 μM). [b] Yields and ee were determined by HPLC using 2‐phenylquinoline as internal standard. For yields <5 % ee's were not determined. All the results listed correspond to the average of two independent experiments, each of them carried out in duplicate. Errors listed are standard deviations. Signs of optical rotation and absolute configuration were assigned by comparison to the literature.^29^, ^30^

2‐methyl‐(1*H*)‐indole (**2 a**) was then combined with β‐phenyl substituted imidazole (**1 b**), resulting in low yields and enantioselectivities of **3 g** with Cu^2+^⊂RamR and Cu^2+^⊂CgmR. However, Cu^2+^⊂QacR led to moderate yield (54 %) of product, which, unlike the other reactions where activity was observed, was similar to that obtained with Cu^2+^ alone. Nevertheless, the product was obtained in 75 % ee, the highest enantioselectivity found in this study, which is clear proof for the protein being involved in the reaction, as it is the only source of chirality. β‐*tert*‐butyl substituted imidazole (**1 c**) resulted in no product formation, regardless of the presence or absence of any of the proteins.

In some cases, the appearance of an extra pair of peaks in the HPLC chromatograms was observed. Based on our previous work, we assigned these peaks to the two enantiomers of the product of conjugate addition of water to the enone (see Figures S6).^30^, ^32^ For the reaction with substrate **3 h**, this was confirmed by comparison of the retention times with those of an independently prepared sample (Figure S14). The formation of this side product was outcompeted by the F−C alkylation reaction where higher yields were obtained, but was more prominent in the cases were F−C reaction yields were lower (see Figures S6).

Overall, among the ArMs investigated here, Cu^2+^⊂QacR was found to be the most active and enantioselective catalyst, except with of 5‐methoxy‐(1*H*)‐indole (**2 d**) where we observed slightly higher activity, but lower selectivity, with Cu^2+^⊂QacR than with Cu^2+^⊂RamR and Cu^2+^⊂CgmR. Furthermore, Cu^2+^⊂QacR, also showed a broader substrate scope compared to Cu^2+^⊂RamR and Cu^2+^⊂CgmR.

The better performance of QacR in catalysis may be related to its high versatility with regard to drug recognition, *i. e*. it binds a wide range of structurally diverse cationic compounds.^24^ QacR's binding flexibility, is believed to be due to several negatively charged residues (Glu57, 58, 90 and 120) within the pocket, responsible for attracting different cationic compounds and neutralizing their charges; as well as to a number of aromatic residues (Trp61, Tyr93, Tyr103, Tyr123 and Phe162) which are involved in the binding of these compounds through general hydrophobic and π‐stacking interactions.

Each binding site of QacR consists of two overlapping mini‐pockets, each of which involves interaction of the drugs with different residues.^24^, ^25^, ^27^ To confirm that these reactions catalyzed by Cu(NO_3_)_2_, in absence of ligand, do take place inside the hydrophobic pocket of the protein and, for example, not on the protein surface, we performed inhibition studies in presence of ethidium bromide, which is known to bind within the pockets of CgmR, RamR and QacR, with reported binding constants of 610 nM,^21^ 14.6 μM^23^ and 2.35 μM,^25^ respectively. We anticipated that ethidium will compete with the substrate‐Cu^2+^ complex for binding into the pockets of the proteins. Since the reaction of **1 a** with **2 a** is protein accelerated, expulsion of the substrate‐Cu^2+^ complex from the pocket by ethidium bromide would cause a decrease in yield and ee. In the case of QacR, the inhibition experiments were also performed with rhodamine 6G, since it is known to bind in a different part of the binding pocket of QacR than ethidium bromide.^26^

Using Cu(NO_3_)_2_ alone, without protein, a small decrease in yield was observed upon addition of the first equivalent of inhibitor, but the yield remained the unchanged upon addition of further equivalents (Table S3). This makes it unlikely that the ethidium bromide or rhodamine 6G directly inhibit the reaction catalyzed by Cu^2+^ alone. In the case of RamR, the presence of ethidium bromide caused a slight decrease in yield but did not affect the enantioselectivity (Figure 2 and Figure S3). In contrast, for CgmR, a decrease in both yield and ee was observed in presence of ethidium bromide. A strong effect was also observed for Cu^2+^⊂QacR. The presence of increasing amounts of both ethidium bromide or rhodamine 6G resulted in a gradual decrease in yield and enantioselectivity (Figure 2). The observation that blocking the binding sites of CgmR and QacR reduced both activity and selectivity of the ArMs, led us to conclude that the catalyzed reaction indeed takes place inside the pockets of these proteins.

**Figure 2:**
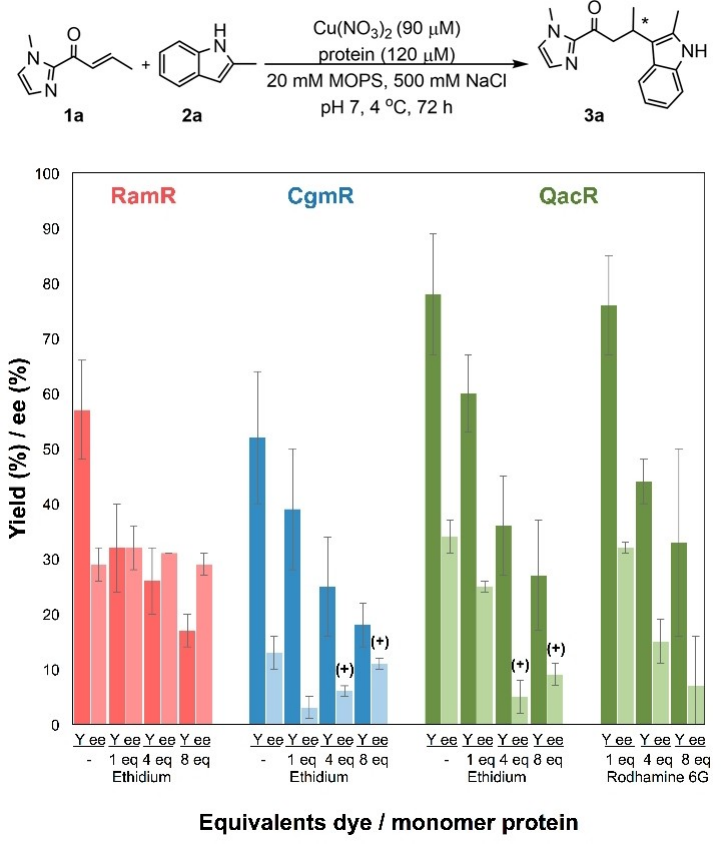
Results of the inhibition experiments for the vinylogous Friedel‐Crafts alkylation reaction. Typical conditions: 90 μM Cu(NO_3_)_2_ (9 mol %) loading with 1.3 eq of protein (120 μM) and indicated amount of dye per monomer of protein. All results correspond to the average of two independent experiments, each carried out in duplicate. Yields (Y) and ee were determined by HPLC using 2‐phenylquinoline as internal standard. Error bars represent standard deviations.

A remarkable result from this study is that, in contrast to our earlier work on LmrR, the TetR based ArMs perform better in catalysis using Cu^2+^ ions alone compared to the Cu(phen) complex. This may be due to the Cu(phen) complex binding in an unfavorable orientation or to the smaller size of the pockets of these proteins compared to LmrR, causing there to be not enough space for the Cu(phen)‐substrate complex. The question then is what is driving the reaction to occur inside the pocket in case of the Cu(NO_3_)_2_ catalyzed reactions? Inspection of the X‐ray crystal structures does not reveal an obvious Cu^2+^ binding site. Thus, it is unlikely that the activated complex is held in place solely by dative anchoring via the Cu^2+^ ion. This is further supported by the observation that assembly of the metalloprotein, followed by dialysis, resulted in a significant drop in activity in the reaction, suggesting the Cu^2+^ ion, in the absence of substrate, is only weakly bound to the protein (Table S4).

Instead, we hypothesize that the highly π‐conjugated and cationic complex formed by Cu^2+^ coordinated to the imidazole substrate (**1**) does have enough affinity to bind in the pockets of these MDR proteins. This occurs without the need of a ligand such as phenanthroline. In this case π‐π stacking interactions and possibly cation π interactions will play a key role. This has precedent in recent work by Onoda, Hayashi and coworkers, who used a pyrene conjugated β‐barrel protein for the catalysis of enantioselective Diels‐Alder reactions by Cu^2+^ salts.^33^ In their study π‐stacking interactions between the substrate and the pyrene moiety were found to be key to the observed catalysis. The available X‐ray crystal structures of these TetR proteins with drugs bound further support this hypothesis: they show that π‐π interactions with phenylalanine and tryptophans play a key role in drug binding.^21^, ^22^, ^26^ Moreover, the pockets of, for example, QacR are rich in negatively charged residues (Glu57, 58, 90 and 120) which can contribute further to the binding of these cationic complexes.

In conclusion, we have shown that CgmR, RamR and QacR, proteins from the TetR family of MDRs, are good scaffolds for the design of ArMs using the supramolecular approach. The side chains of amino acids located in their pockets create networks of hydrophobic interactions that allow the formation of ArMs for the enantioselective vinylogous Friedel‐Crafts alkylation reaction using Cu^2+^ ions and an aromatic substrate without the need of an additional ligand for the metal ion. This approach constitutes a fast and straightforward way to assemble metalloenzymes which provides significant advantages compared to standard supramolecularly‐assembled ArMs, as it avoids the need for an external ligand for the metal ion. This circumvents also the need for a ligand‐screening step while designing new ArMs and it facilitates optimization of the protein scaffold via mutagenesis or directed evolution approaches.

## Conflict of interest

The authors declare no conflict of interest.

## Supporting information

As a service to our authors and readers, this journal provides supporting information supplied by the authors. Such materials are peer reviewed and may be re‐organized for online delivery, but are not copy‐edited or typeset. Technical support issues arising from supporting information (other than missing files) should be addressed to the authors.

Supplementary
