# Supplementary material for: Artificial Metalloenzymes based on TetR Proteins and Cu(II) for Enantioselective Friedel‐Crafts Alkylation Reactions
Source: ChemCatChem. 2020 Apr 29;12(12):3190–4. doi: 10.1002/cctc.202000245 (PMC7319431; doi:10.1002/cctc.202000245)
Supplement: Supplementary file 1 — Supplementary [file CCTC-12-3190-s001.pdf]

ChemCatChem

Supporting Information

# Artificial Metalloenzymes based on TetR Proteins and Cu(II) for Enantioselective Friedel-Crafts Alkylation Reactions

Cora Gutiérrez de Souza, Manuela Bersellini, and Gerard Roelfes*© 2020 The Authors. Published by Wiley-VCH Verlag GmbH & Co. KGaA. This is an open access article under the terms of the Creative Commons Attribution License, which permits use, distribution and reproduction in any medium, provided the original work is properly cited.

## Contents

1. Materials and methods ........................................................................................... 2

2. Synthetic procedures and characterization............................................................ 2

3. Molecular biology ............................................................................................ 15

4. Protein production, purification and characterization..................................... 165

5. UV-visible titrations......................................................................................... 211

6. Catalysis: Friedel-Crafts alkylation ................................................................... 233

7. References ........................................................................................................ 422

## 1. Materials and methods

All the chemicals and reagents were purchased from commercial suppliers (Sigma Aldrich or Acros) and used without further purification. Flash chromatography was performed using Reveleris® X2 Flash Chromatography System. $^{1}$H-NMR and $^{13}$C-NMR spectra were recorded on a Varian 400 (400 MHz) spectrometer in $CDCl_3$. Chemical shifts values (δ) are denoted in ppm using residual solvent peaks as the internal standard ($CHCl_3$: δ 7.26 for 1H). $^{1}$H-NMR results are reported in the conventional form: chemical shifts, multiplicity (br = broad, d = doublet, dd = doublet of doublet, ddd = doublet of doublet of doublets, dq=doublet of quartets, m=multiplet, q=quartet, s=singlet, t=triplet), coupling constants (Hz), and integration. Data for $^{13}$C-NMR are reported in terms of chemical shift (δ ppm) and multiplicity. Enantiomeric excess determinations were performed by HPLC analysis using UV-detection (Shimadzu SCL-10Avp) on Chiralpak AD, n-heptane:iPrOH 90:10, 1.0 ml/min. UPLC-MS on protein samples was performed on a Acquity TQ Detector (ESITQD-MS) coupled to Waters Acquity Ultra Performance LC using a Acquity BEH C8 (1.7 µm 2.1 x 150 mm). Water (solvent A) and acetonitrile (solvent B) containing 0.1% formic acid by volume, were used as the mobile phase at a flow rate of 0.3 mL/min. Gradient: 90% A for 2 min, linear gradient to 50% A in 2 min, linear gradient to 20% A in 5 min, followed by 2 min at 5% A. Re-equilibration of the column with 2 min at 90% A. E. coli strain NEB5α-T1 Phage Resistant (Stratagene) was used for cloning and E. coli BL21(DE3)-C43-T1 Phage Resistant (Stratagene) strain was uesd for protein expression. Primers were synthesized by Eurofins MWG Operon (Ebesberg, Germany). Pfu Turbo polymerase was purchased from Agilent and DpnI was purchased from New England Biolabs. Plasmid isolation Kit was purchased from Roche. DNA sequencing was carried out by Eurofins Genomics. FPLC columns were purchased from GE Healthcare. Size exclusion chromatography was performed using AKTApurifier 10 apparatus. The concentration of the proteins was measured with Nanodrop 2000 (Thermo Scientific).

## 2. Synthetic procedures and characterization

### General procedure for the preparation of the Friedel-Crafts products as reference material

Procedure was adapted from literature.[1,2] 50 mg of the corresponding α,β-unsaturated-2-acyl imidazole and 2 equivalents of the corresponding indole, predissolved in 10 mL acetonitrile, were added to 1 L of water containing 2.3 g sodium dodecyl sulfate (8 mM final concentration) and 15 mol% $Cu(NO_3)_2 \cdot 3H_2O$.The reaction was stirred at room temperature for 16 hours. 10 g of NaCl was added and the aqueous phase was extracted 3 times with 100 mL diethyl ether. Subsequently, the organic phase was washed with 100 mL brine and dried on $Na_2SO_4$. The product was purified by column chromatography with a gradient heptane:ethyl acetate.

Some of the reference products present a peak at 7.26 ppm corresponding to benzene, which was used as internal standard to calculate the amount of impurity present in the sample and correct for it.

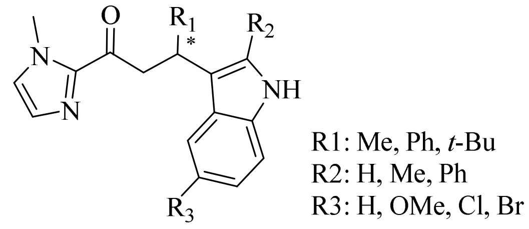

**3-(1H-indol-3-yl)-1-(1-methyl-1H-imidazol-2-yl)butan-1-one (3a)**[3,4]

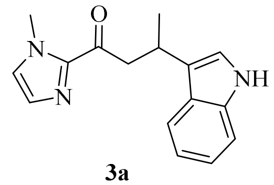


**3a**

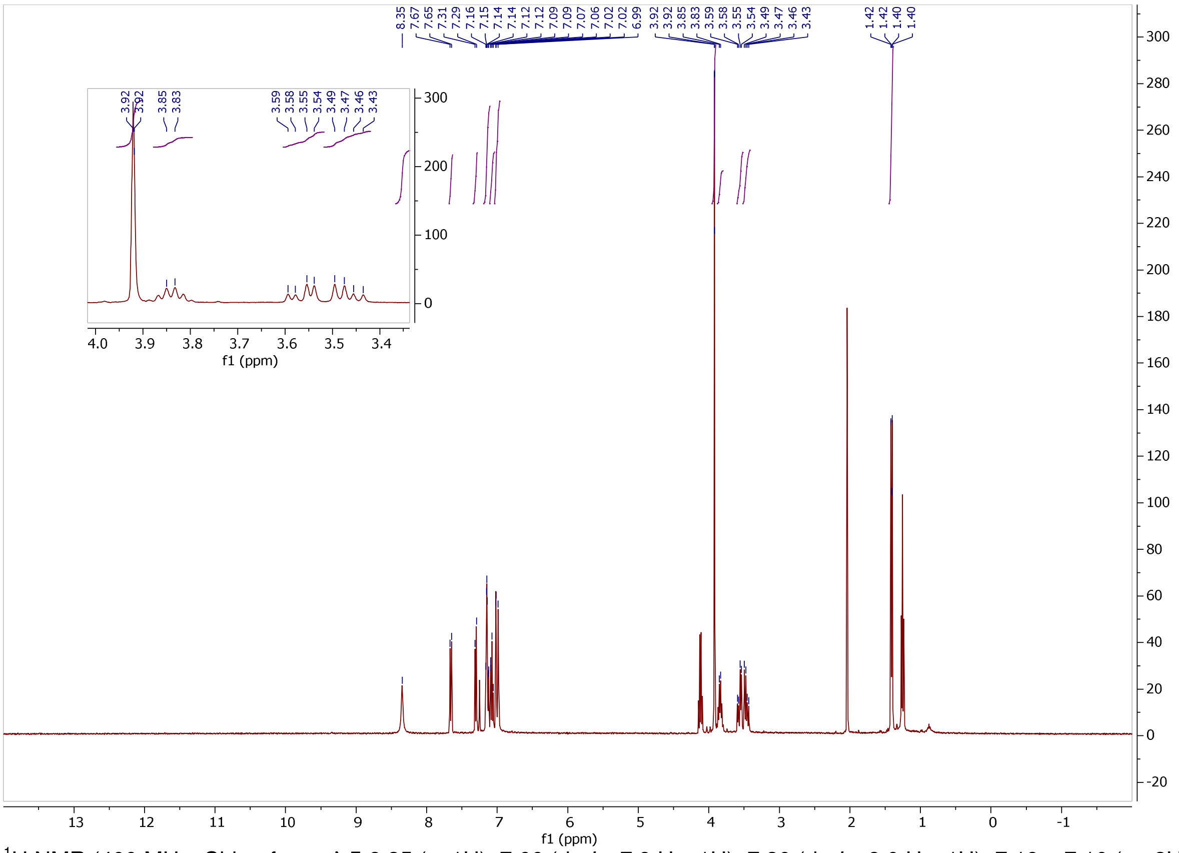


$^{1}$H NMR (400 MHz, Chloroform-*d*) δ 8.35 (s, 1H), 7.66 (d, *J* = 7.9 Hz, 1H), 7.30 (d, *J* = 8.0 Hz, 1H), 7.19 – 7.10 (m, 2H), 7.10 – 7.04 (m, 1H), 7.04 – 6.96 (m, 2H), 3.92 (d, *J* = 1.2 Hz, 3H), 3.84 (d, *J* = 7.1 Hz, 1H), 3.57 (dd, *J* = 15.9, 6.3 Hz, 1H), 3.46 (dd, *J* = 15.9, 8.2 Hz, 1H), 1.41 (dd, *J* = 6.9, 1.3 Hz, 3H).

**1-(1-methyl-1H-imidazol-2-yl)-3-(2-methyl-1H-indol-3-yl)butan-1-one (3b)**[4–6]

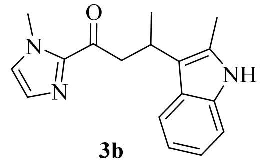


**3b**

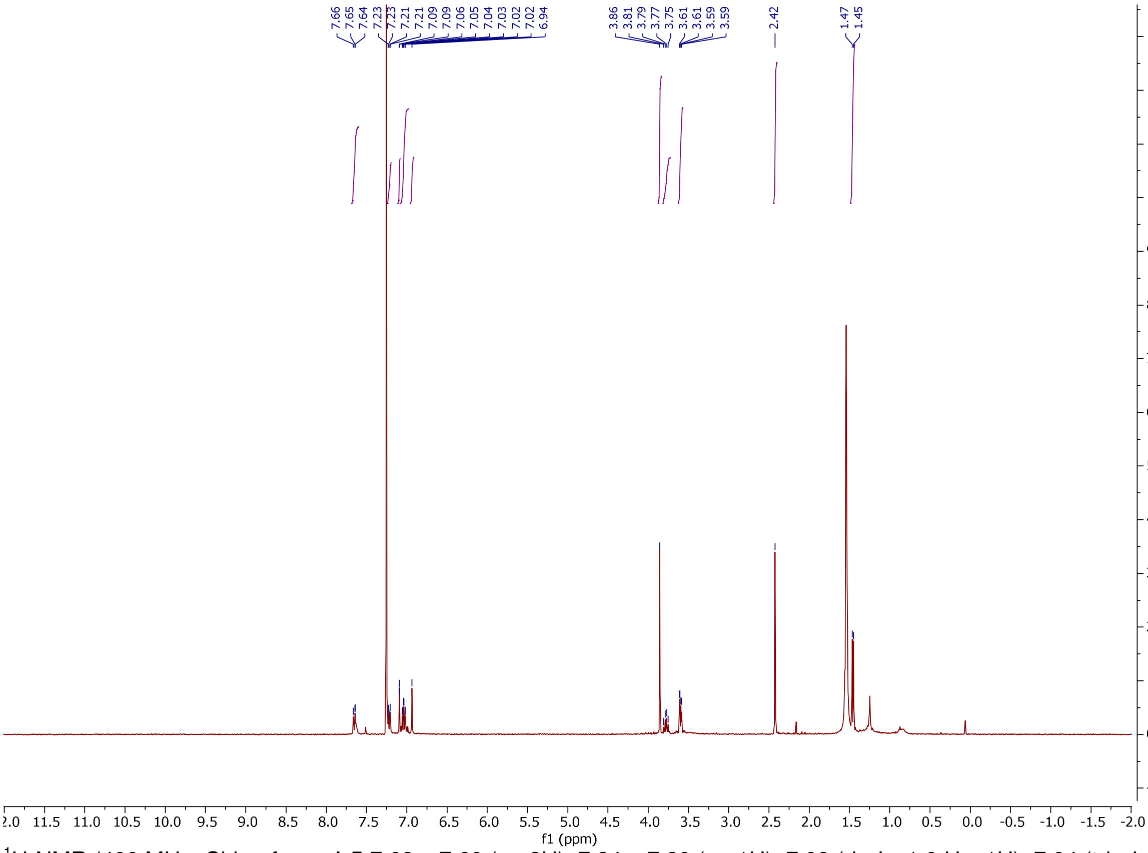


$^{1}$H-NMR (400 MHz, Chloroform-*d*) δ 7.68 – 7.60 (m, 2H), 7.24 – 7.20 (m, 1H), 7.09 (d, *J* = 1.0 Hz, 1H), 7.04 (td, *J* = 6.9, 1.5 Hz, 2H), 6.94 (s, 1H), 3.86 (s, 3H), 3.78 (m, *J* = 7.2 Hz, 1H), 3.60 (dd, *J* = 7.4, 2.8 Hz, 2H), 2.42 (s, 3H), 1.46 (d, *J* = 7.1 Hz, 3H).

**1-(1-methyl-1H-imidazol-2-yl)-3-(2-phenyl-1H-indol-3-yl)butan-1-one (3c)**

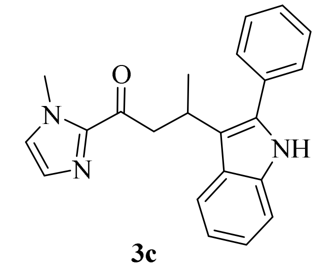


**3c**

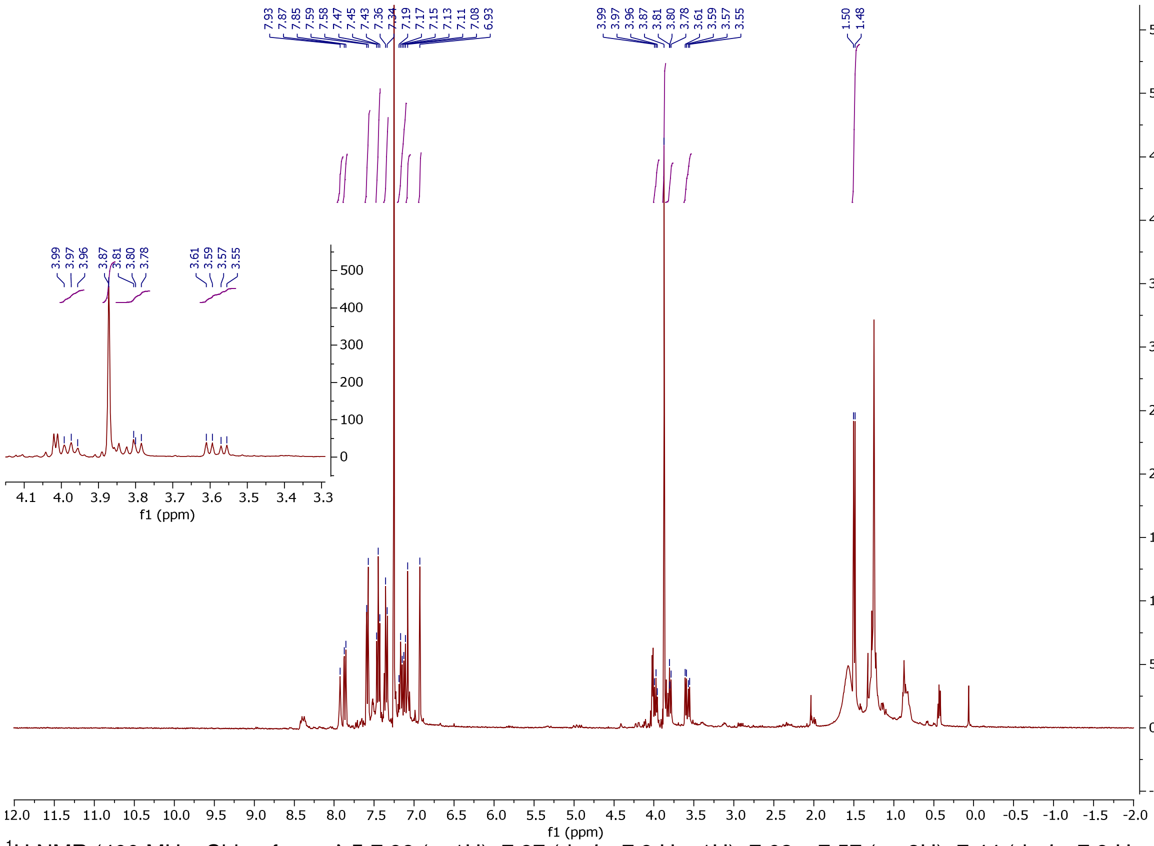


$^{1}$H NMR (400 MHz, Chloroform-*d*) δ 7.98 (s, 1H), 7.87 (d, *J* = 7.9 Hz, 1H), 7.62 – 7.57 (m, 3H), 7.44 (d, *J* = 7.9 Hz, 2H), 7.40 – 7.31 (m, 2H), 7.21 – 7.06 (m, 2H), 6.93 (s, 1H), 4.02 – 3.94 (m, 1H), 3.88 (s, 3H), 3.81 (dd, *J* = 15.8, 8.5 Hz, 1H), 3.58 (dd, *J* = 15.8, 6.3 Hz, 1H), 1.49 (d, *J* = 7.0 Hz, 3H). HRMS (ES+): Exact mass calcd for $C_{22}H_{21}N_3O$ $[M+H]^+$ 343.1684, found 342.1607.

**3-(5-methoxy-1H-indol-3-yl)-1-(1-methyl-1H-imidazol-2-yl)butan-1-one (3d)**[2,5,6]

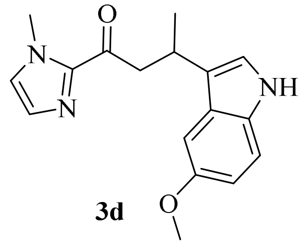

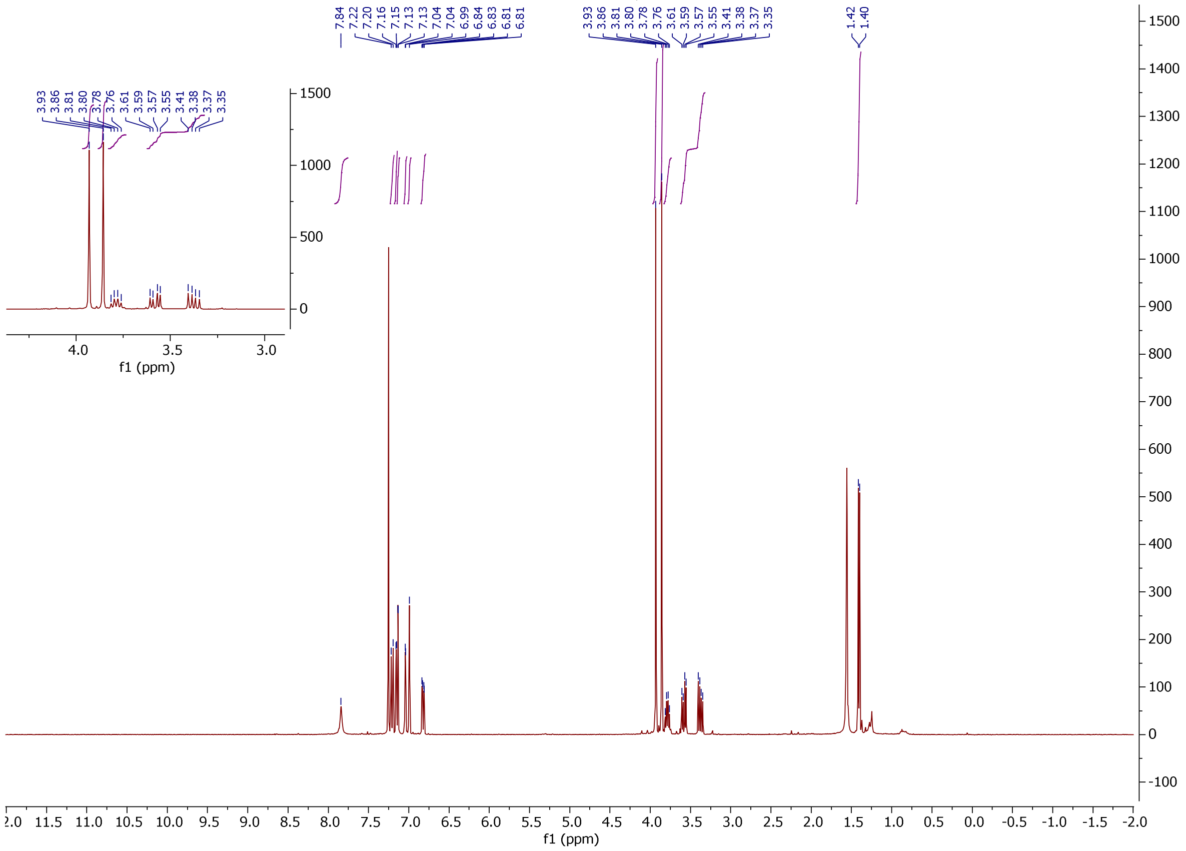


$^1$H-NMR (400 MHz, Chloroform-*d*) δ 7.84 (s, 1H), 7.21 (d, *J* = 8.8 Hz, 1H), 7.16 (d, *J* = 2.4 Hz, 1H), 7.14 – 7.12 (m, 1H), 7.04 (d, *J* = 2.4 Hz, 1H), 6.99 (s, 1H), 6.82 (dd, *J* = 8.8, 2.4 Hz, 1H), 3.93 (s, 3H), 3.86 (s, 3H), 3.79 (m, *J* = 7.0 Hz, 1H), 3.62 – 3.32 (m, 2H), 1.41 (d, *J* = 6.9 Hz, 3H).

**3-(5-chloro-1H-indol-3-yl)-1-(1-methyl-1H-imidazol-2-yl)butan-1-one (3e)**[4–6]

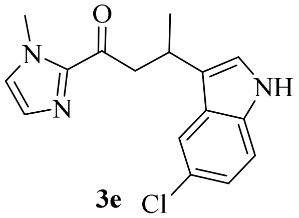


**3e**

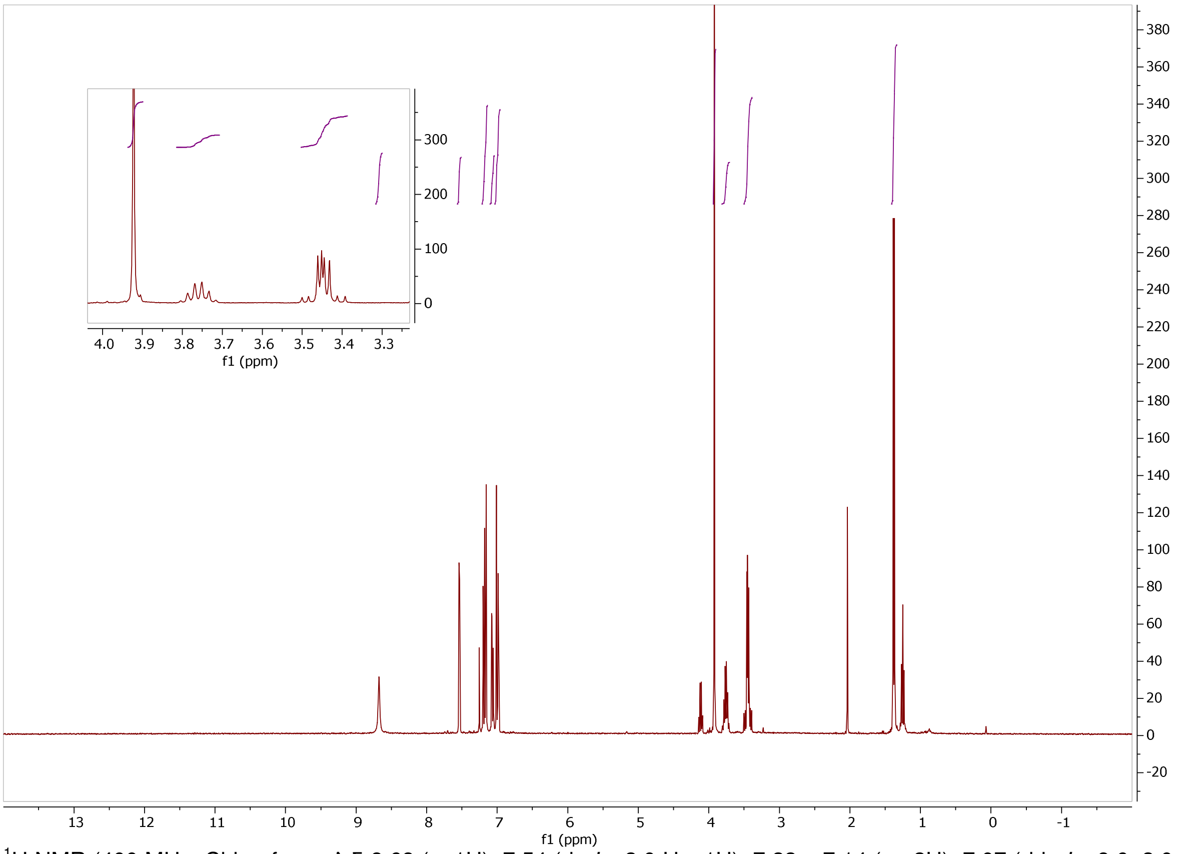


$^{1}$H NMR (400 MHz, Chloroform-*d*) δ 8.68 (s, 1H), 7.54 (d, *J* = 2.0 Hz, 1H), 7.22 – 7.14 (m, 2H), 7.07 (dd, *J* = 8.6, 2.0 Hz, 1H), 7.03 – 6.96 (m, 2H), 3.92 (s, 3H), 3.81 – 3.71 (m, 1H), 3.50 – 3.39 (m, 2H), 1.38 (d, *J* = 6.9 Hz, 3H).

**3-(5-bromo-1H-indol-3-yl)-1-(1-methyl-1H-imidazol-2-yl)butan-1-one (3f)**

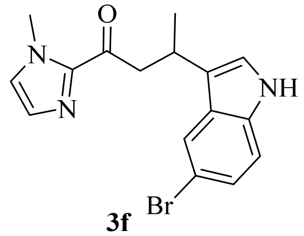


**3f**

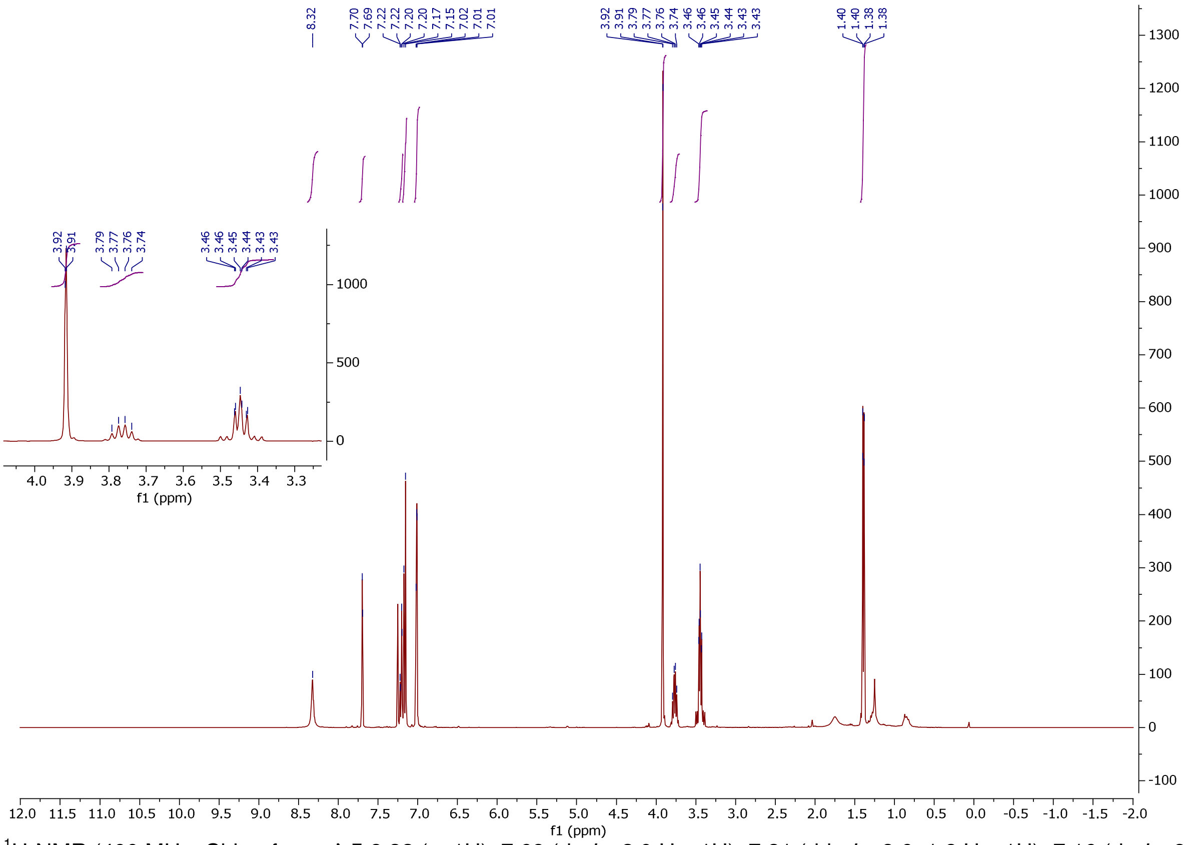


$^{1}$H-NMR (400 MHz, Chloroform-*d*) δ 8.32 (s, 1H), 7.69 (d, *J* = 2.0 Hz, 1H), 7.21 (dd, *J* = 8.6, 1.8 Hz, 1H), 7.16 (d, *J* = 8.6 Hz, 2H), 7.04 – 6.98 (m, 2H), 3.92 (d, *J* = 1.3 Hz, 3H), 3.77 (m, *J* = 7.1 Hz, 1H), 3.44 (m, *J* = 6.2, 1.2 Hz, 2H), 1.39 (dd, *J* = 6.9, 1.2 Hz, 3H). $^{13}$C-NMR (101 MHz, Chloroform-*d*) δ 194.6, 145.8, 137.5, 131.5, 131.0, 129.6, 127.3, 124.4, 124.1, 123.8, 115.1, 115.0, 49.6, 38.8, 29.7, 24.2.

**1-(1-methyl-1H-imidazol-2-yl)-3-(2-methyl-1H-indol-3-yl)-3-phenylpropan-1-one (3g)**[6]

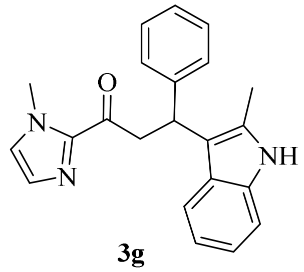

**3g**

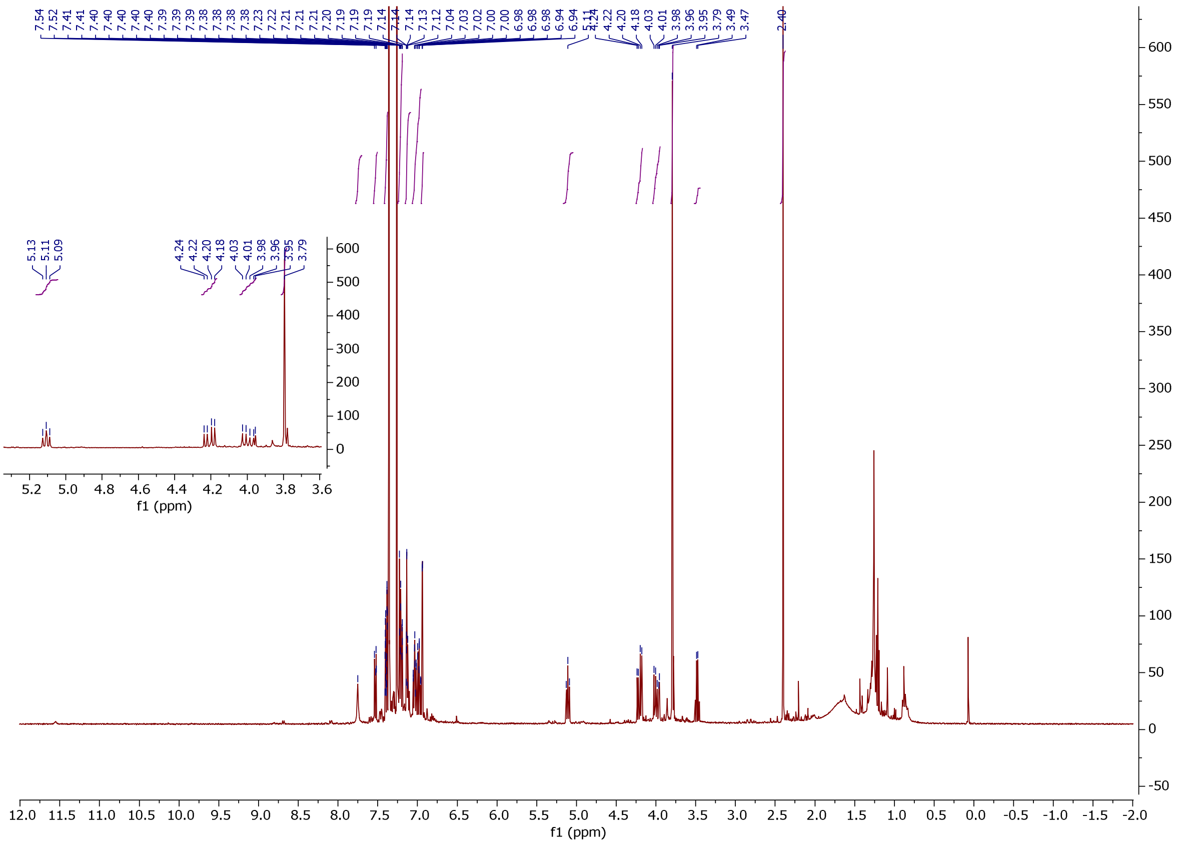

$^1$H-NMR (400 MHz, Chloroform-*d*) δ 7.75 (s, 1H), 7.55 – 7.50 (m, 1H), 7.42 – 7.38 (m, 2H), 7.25 – 7.19 (m, 3H), 7.16 – 7.09 (m, 2H), 7.07 – 6.95 (m, 2H), 6.94 (d, *J* = 1.0 Hz, 1H), 5.11 (t, *J* = 7.6 Hz, 1H), 4.21 (dd, *J* = 16.6, 7.0 Hz, 1H), 4.04 – 3.95 (m, 1H), 3.79 (s, 3H), 2.40 (s, 3H).

**4,4-dimethyl-1-(1-methyl-1H-imidazol-2-yl)-3-(2-methyl-1H-indol-3-yl)pentan-1-one (3h)**

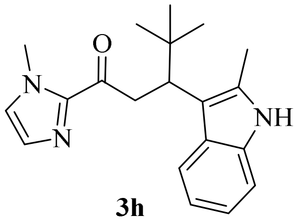


**3h**

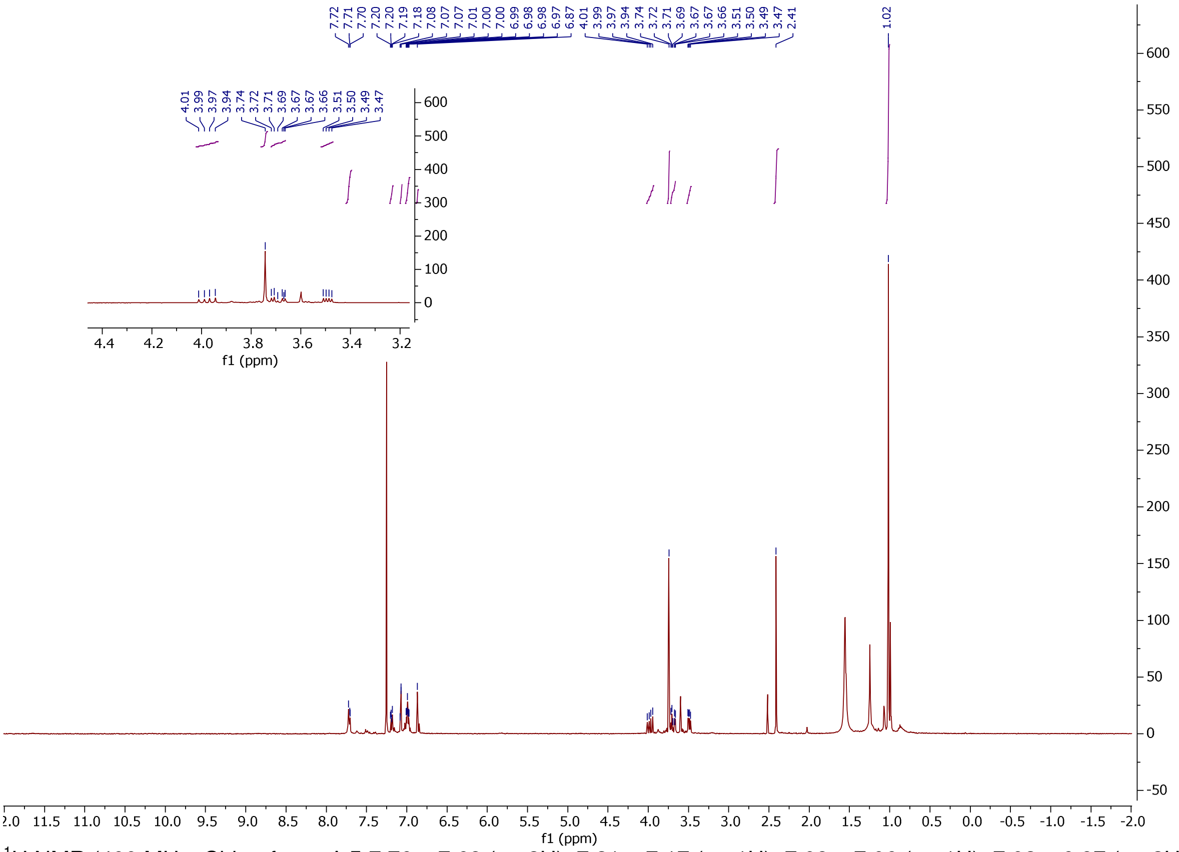


$^{1}$H NMR (400 MHz, Chloroform-*d*) δ 7.76 – 7.68 (m, 2H), 7.21 – 7.17 (m, 1H), 7.08 – 7.06 (m, 1H), 7.02 – 6.97 (m, 2H), 6.87 (s, 1H), 3.98 (dd, *J* = 17.6, 9.1 Hz, 1H), 3.74 (s, 3H), 3.72 – 3.66 (m, 1H), 3.49 (dd, *J* = 9.1, 4.6 Hz, 1H), 2.41 (s, 3H), 1.02 (s, 9H).

## General procedure for the preparation of the Friedel-Crafts substrate

**(E)-1-(1-Methyl-1H-imidazole-2-yl)-but-2-en-1-one (1a)** was synthesized according to a literature procedure.[3,5] Starting from 861 mg of crotonic acid (10 mmol) **1a** was obtained in a 20% purified yield (300 mg).

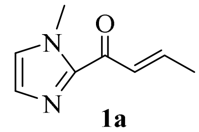


**1a**

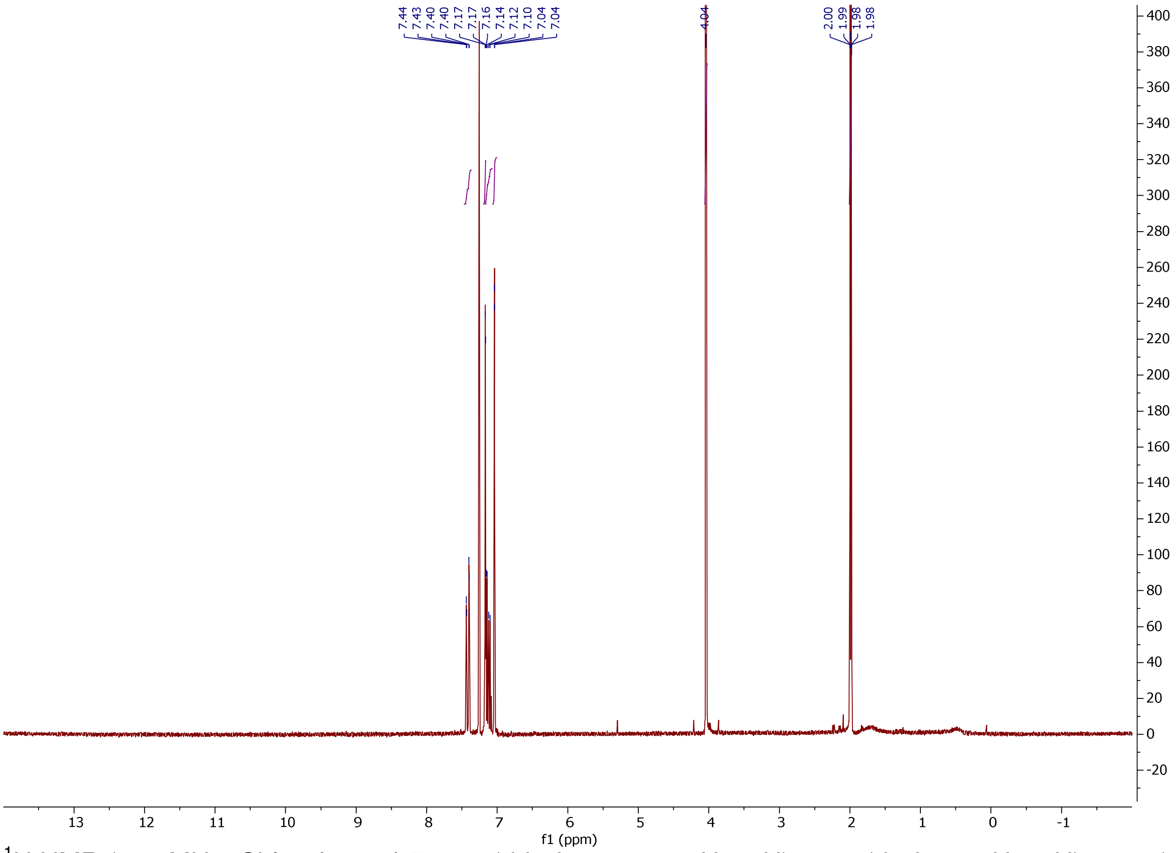


$^{1}$H NMR (400 MHz, Chloroform-*d*) δ 7.42 (dd, *J* = 15.5, 1.7 Hz, 1H), 7.17 (d, *J* = 1.0 Hz, 1H), 7.13 (dd, *J* = 15.5, 6.9 Hz, 1H), 7.04 (d, *J* = 0.9 Hz, 1H), 4.04 (s, 3H), 1.99 (dd, *J* = 6.9, 1.7 Hz, 3H).

**(E)-1-(1-methyl-1H-imidazol-2-yl)-3-phenylprop-2-en-1-one (1b)**[3]

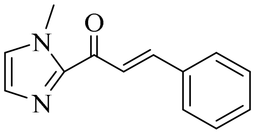

**1b**

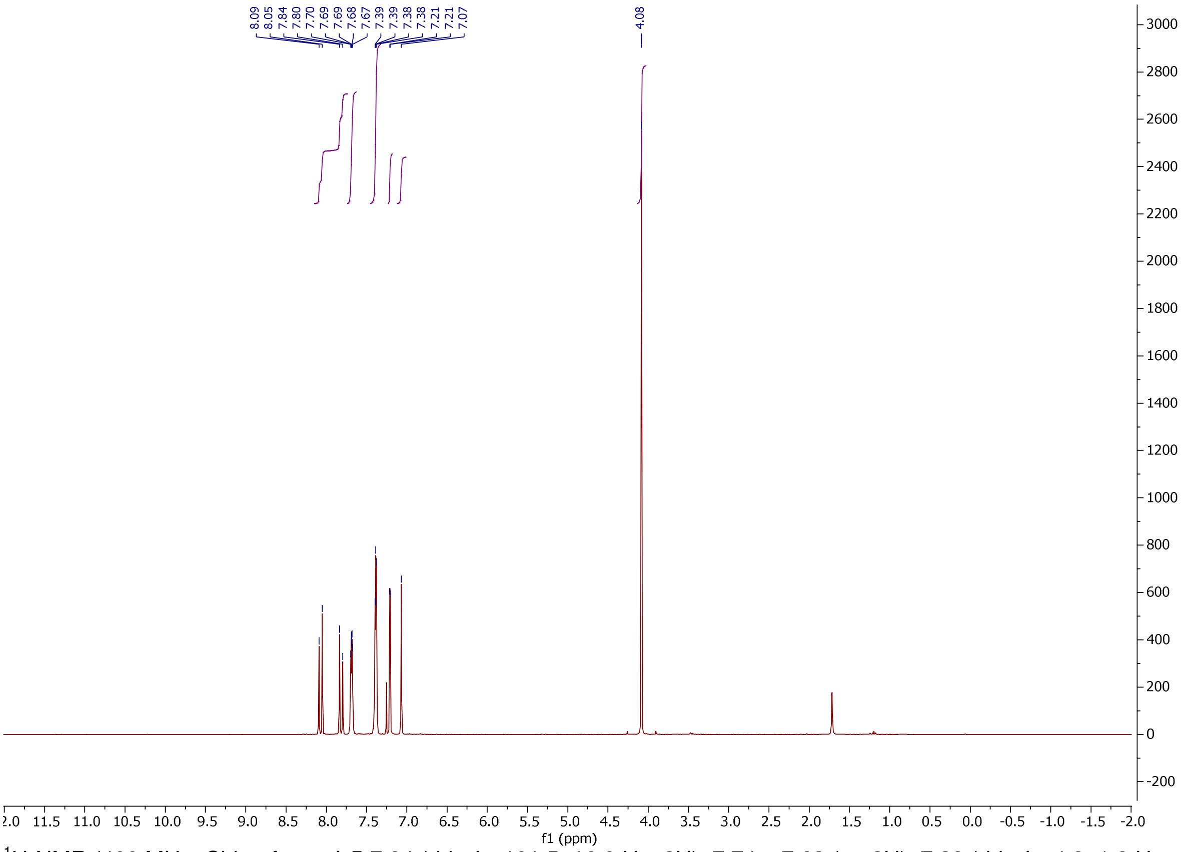

$^{1}$H-NMR (400 MHz, Chloroform-*d*) δ 7.94 (dd, *J* = 101.5, 16.0 Hz, 2H), 7.74 – 7.63 (m, 2H), 7.39 (dd, *J* = 4.9, 1.9 Hz, 3H), 7.21 (d, *J* = 1.0 Hz, 1H), 7.07 (s, 1H), 4.08 (s, 3H).

**(E)-4,4-dimethyl-1-(1-methyl-1H-imidazol-2-yl)pent-2-en-1-one (1c)**[7]

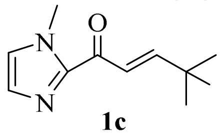

**1c**

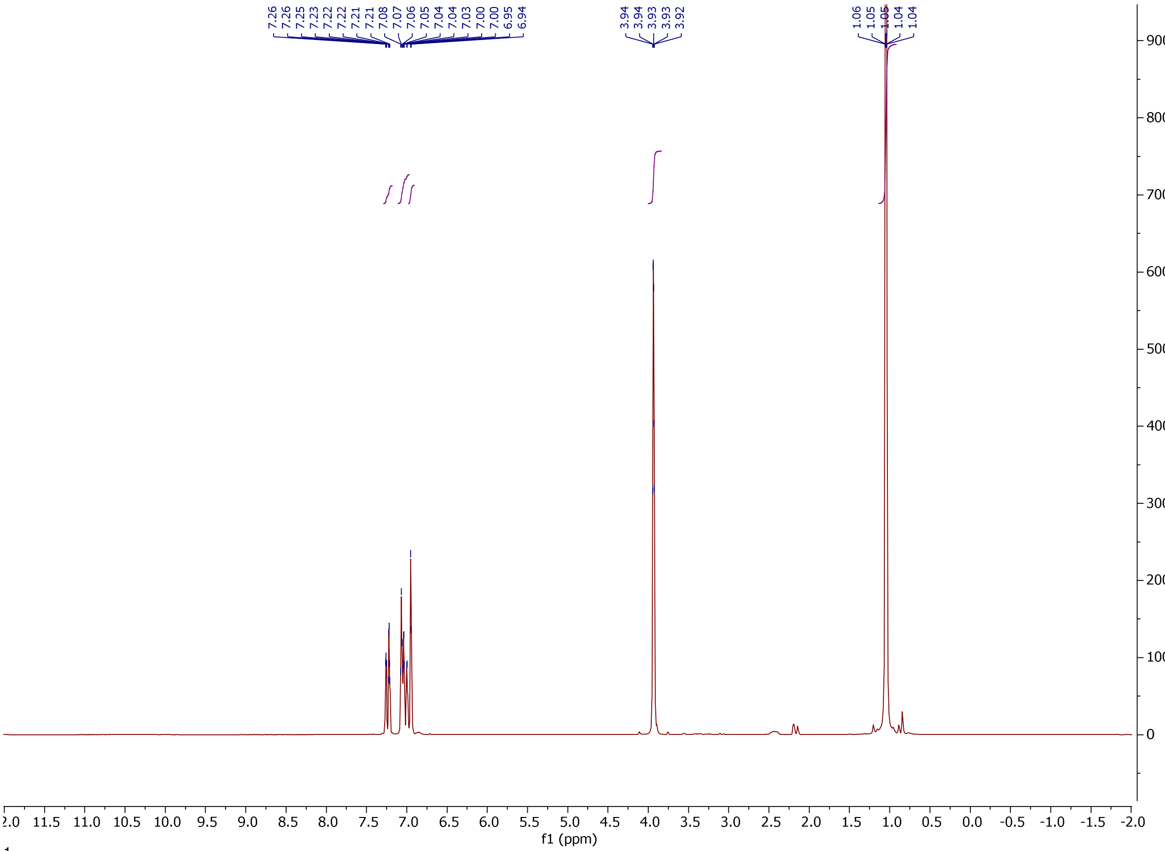


$^1$H-NMR (400 MHz, Chloroform-*d*) δ 7.29 – 7.18 (m, 1H), 7.11 – 6.97 (m, 2H), 6.95 (d, *J* = 3.7 Hz, 1H), 3.93 (s, 3H), 1.05 (s, 9H).

## Water-addition product

### 3-hydroxy-4,4-dimethyl-1-(1-methyl-1H-imidazol-2-yl)pentan-1-one (4)

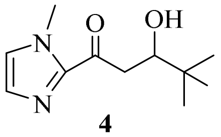

**4**

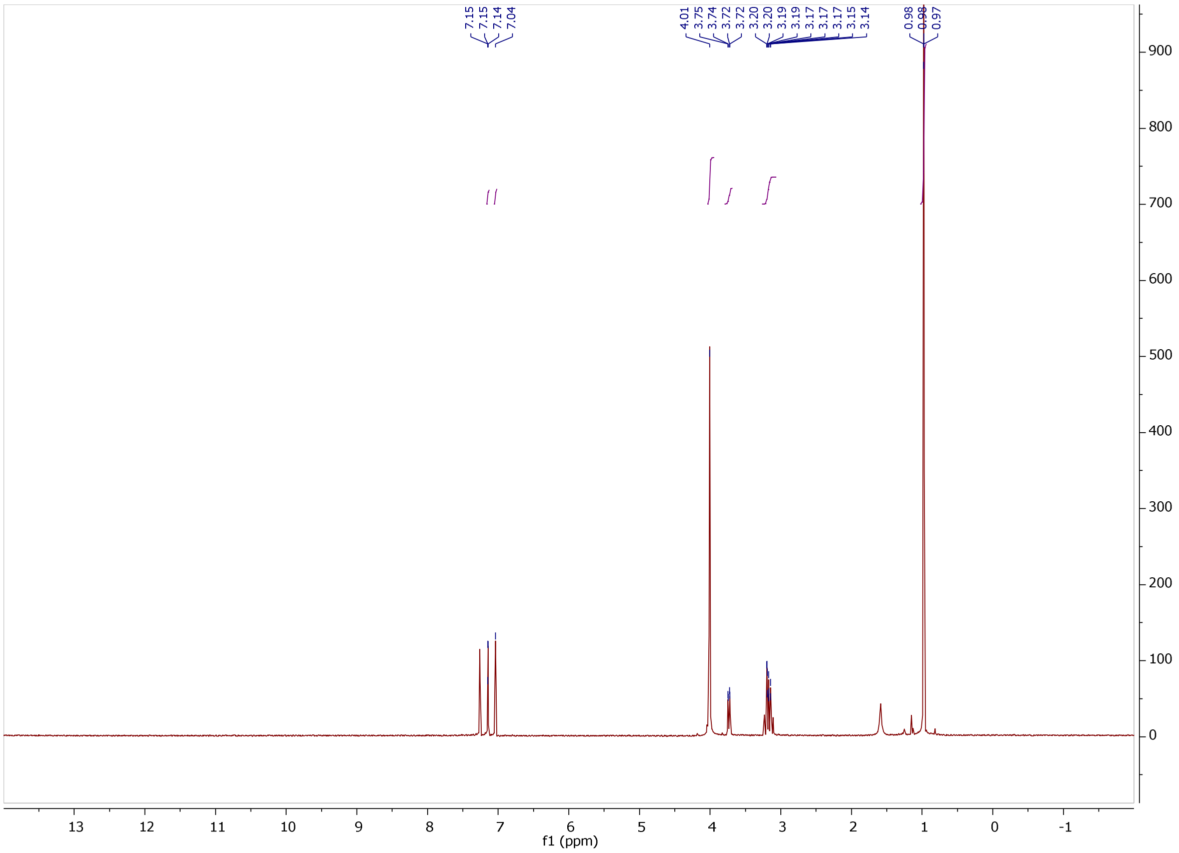

1H-NMR (400 MHz, Chloroform-d) δ 7.16 – 7.13 (s, 1H), 7.04 (s, 1H), 4.01 (s, 3H), 3.73 (dd, J = 9.4, 2.2 Hz, 1H), 3.26 – 3.07 (m, 2H), 0.98 (s, 9H).

## 3. Molecular biology

pET 17b plasmids encoding for wt-QacR ,wt-CgmR and wt-RamR were available from a previous work.[4] Plasmids were purchased from Genescript (USA) as codon optimized sequences for E.Coli expression and included a C-terminal Strep-tag for purification purposes. Gene sequences:

**RamR**, Codon optimized: Host expression organism: Escherichia coli, Length: 624 bp,
CATATGGTGGCGCGTCCGAAGAGCGAGGACAAGAAACAAGCGCTGCTGGAAGCGGCGACCCAGGCGATTGCGCAAAGCGGTATT
GCGGCGAGCACCGCGGTGATTGCGCGTAACGCGGGTGTTGCGGAGGGTACCCTGTTCCGTTACTTTGCGACCAAGGACGAACTGA
TTAACACCCTGTATCTGCACCTGAAACAGGATCTGAGCCAAAGCATGATCATGGAGCTGGACCGTAGCATTACCGATGCGAAAATGA
TGACCCGTTTCATCTGGAACAGCTACATTAGCTGGGGCCTGAACCATCCGGCGCGTCACCGTGCGATCCGTCAGCTGGCGGTTAG
CGAGAAGCTGACCAAAGAAACCGAACAACGTGCGGACGATATGTTCCCGGAACTGCGTGATCTGAGCCACCGTAGCGTGCTGATG
GTTTTTATGAGCGACGAGTACCGTGCGTTCGGTGATGGCCTGTTTCTGGCGCTGGCGGAAACCACCATGGATTTTGCGGCGCGTGA
TCCGGCGCGTGCGGGCGAGTATATTGCGCTGGGCTTTGAAGCGATGTGGCGTGCGCTGACCCGTGAGGAACAGGCGGCGTGGAG
CCACCCGCAATTTGAAAAGTAACTCGAG

**QacR**, Codon optimized: Host expression organism: Escherichia coli, Length: 606 bp,
CATATGAACCTGAAGGACAAAATCCTGGGTGTGGCGAAGGAGCTGTTCATTAAAAACGGTTATAACGCGACCACCACCGGCGAGAT
CGTTAAGCTGAGCGAAAGCAGCAAAGGCAACCTGTACTATCACTTCAAGACCAAAGAGAACCTGTTTCTGGAAATCCTGAACATTGA
GGAAAGCAAGTGGCAGGAGCAATGGAAAAGCGAACAGATTAAGTGCAAAACCAACCGTGAGAAGTTCTATCTGTACAACGAACTGA
GCCTGACCACCCAGTACTATTACCCGCTGCAAAACGCGATCATCGAGTTCTACACCGAATACTACAAGACCAACAGCATCAACGAGA
AGATGAACAAACTGGAAAACAAGTATATCGACGCGTACCACGTGATTTTCAAAGAGGGTAACCTGAACGGCGAATGGTGCATTAACG
ATGTGAACGCGGTTAGCAAGATCGCGGCGAACGCGGTGAACGGTATTGTTACCTTTACCCACGAGCAGAACATCAACGAACGTATT
AAGCTGATGAACAAATTCAGCCAAATCTTTCTGAACGGCCTGAGCAAGGCGGCGTGGAGCCACCCGCAATTTGAAAAATAACTCGA
G

**CgmR**, Codon optimized: Host expression organism: Escherichia coli, Length: 573 bp,
CATATGCGTACCAGCAAGAAAGAGATGATCCTGCGTACCGCGATCGACTACATTGGCGAGTATAGCCTGGAAACCCTGAGCTACGA
TAGCCTGGCGGAAGCGACCGGTCTGAGCAAGAGCGGCCTGATCTATCACTTTCCGAGCCGTCATGCGCTGCTGCTGGGTATGCAC
GAGCTGCTGGCGGACGATTGGGACAAAGAACTGCGTGATATTACCCGTGACCCGGAGGATCCGCTGGAACGTCTGCGTGCGGTGG
TTGTGACCCTGGCGGAGAACGTTAGCCGTCCGGAACTGCTGCTGCTGATTGATGCGCCGAGCCACCCGGATTTCCTGAACGCGTG
GCGTACCGTGAACCACCAGTGGATTCCGGACACCGACGATCTGGAGAACGATGCGCACAAGCGTGCGGTTTACCTGGTGCAACTG
GCGGCGGACGGTCTGTTTGTTCACGATTATATTCACGACGATGTGCTGAGCAAGAGCAAACGTCAGGCGATGCTGGAAACCATCCT
GGAACTGATTCCGAGCGAGCTGGCGGCGTGGAGCCACCCGCAATTCGAAAAATAACTCGAG

## 4. Protein production, purification and characterization

pET17b plasmids encoding for wt-CgmR, wt-RamR and QacR_C72A_C141S, were transformed into E. coli BL21(DE3)C43 cells. The cells were spread onto an agar plate containing 100 µg/mL of ampicillin. 5 mL of LB media with µg/mL of ampicillin were inoculated with a single colony from the transformation and grown at 37 °C overnight. The entire starter culture was used to inoculate 500 mL of fresh LB medium containing 100 µg/mL of ampicillin. When the culture reached an optical density at 600 nm between 0.8-0.9, isopropyl β-D-1thiogalactopyranoside (IPTG) at a final concentration of 1 mM was added to induce the expression of the target proteins. Protein expression was performed at 30 ºC overnight. Cells were harvested by centrifugation (6000 rpm, JA-10, 20 min, 4 ºC, Beckman) and the pellet was resuspended in 50 mM $NaH_2PO_4$, pH 8.0, 150 mM NaCl and protease inhibitor cocktail (Complete, Roche, 1 tablet, for 50 mL of resuspension buffer). Cells were sonicated (70% (200 W) for 15 min (10 sec on, 10 sec off) and afterwards incubated for 30 min on ice with PMSF solution (final concentration 0.1 mM), DNaseI (0.1 mg/mL) and 10 mM $MgCl_2$. After centrifugation (16000 rpm, JA-17, 45 min, 4°C, Beckman), the supernatant was equilibrated with 5 mL of pre-equilibrated Strep-tag Tactin column material for 1 h (mixed at 200 rpm on a rotatory shaker) at 4°C. The column was washed with three times with 3XCV (column volume) of the re-suspension buffer, and the protein was eluted with five 1X CV of resuspension buffer containing 5 mM desthiobiotin. Elution fractions were analyzed on a 12% polyacrylamide SDS-Tris Tricine gel followed by Coumassie staining (InstantBlue, Expedeon). Fractions containing protein were pulled concentrated using Vivaspin Turbo (5000 MWCO, Sartorius) centrifugation filters and they were dialyzed twice against 20 mM MOPS, 500 mM NaCl, pH 7 buffer. Concentration of the proteins was measured on a Nanodrop 2000 (Thermo Scientific), using the calculated extinction coefficient for the monomer (Protparam, Expasy server). The proteins with $A^{260}/A^{280}$ higher than 0.7, were purified *via* cation exchange chromatography on a Hitrap Heparin HP column by a gradient of NaCl concentration from 0 to 1 M in 5 min with a flow of 1 mL $min^{-1}$. Typical expression yields were between 15 and 30 mg/L. Analytical size exclusion chromatography was performed on a Superdex 75 10/300 GL (GE Healthcare). 100 µL of the sample was injected using 20 mM MOPS, pH 7.0, 500 mM NaCl as buffer (flow 0.5 mL $min^{-1}$).

**Figure S1. SDS-PAGE after Strep-Tag purification**

**QacR_C72A_C141S**

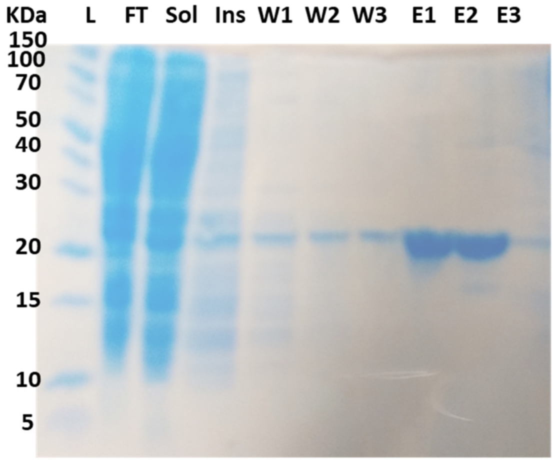


**RamR**

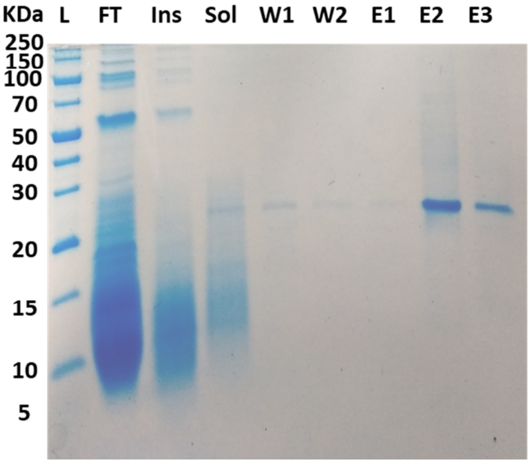


**CgmR**

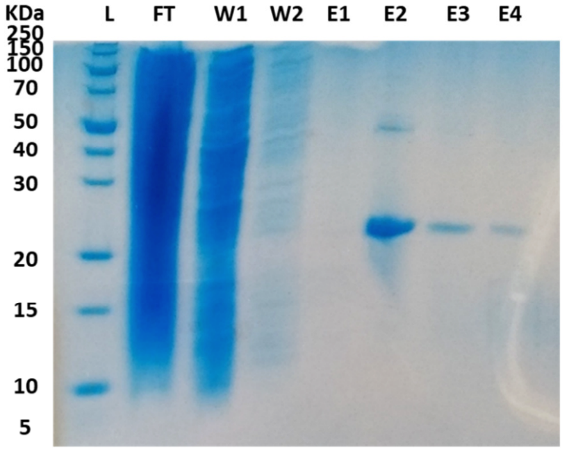


L: protein ladder (Unstained PageRuler Broad Range Protein Ladder), FT: flow through, sol: soluble fraction; ins: insoluble fraction W: wash fraction; E: elution fraction

**Figure S2. Heparin purification chromatography traces**

**QacR_C72A_C141S**

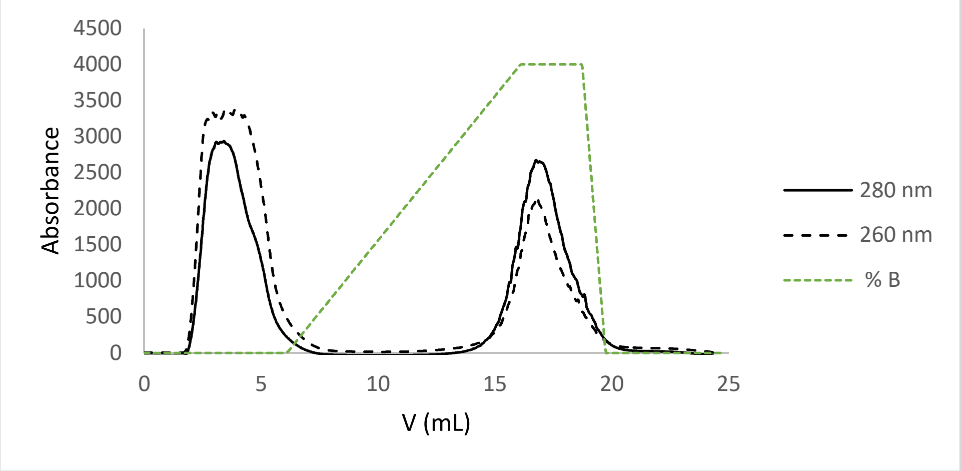


**RamR**

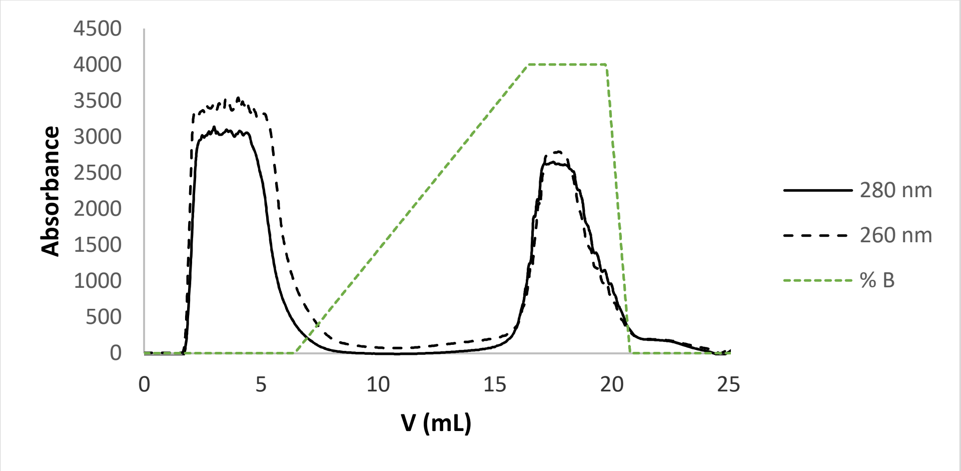


**CgmR**

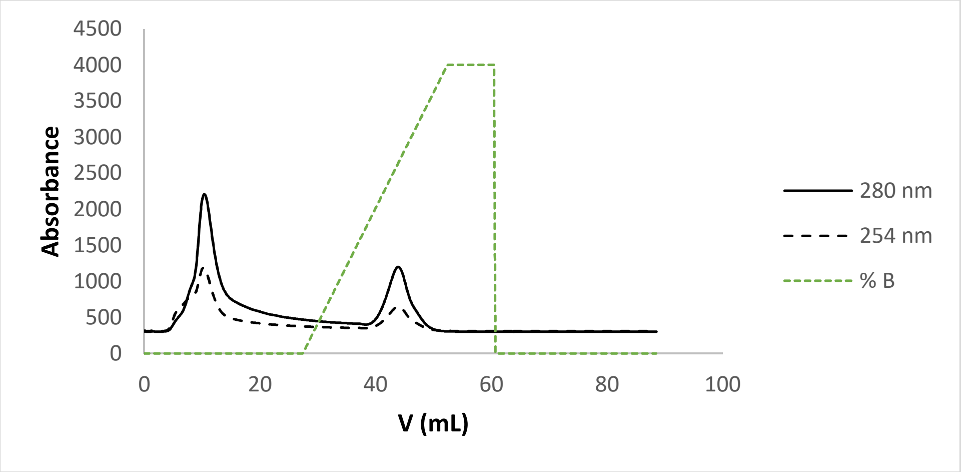

**Figure S3. Mass spectra from UPLC-MS ESI(+) analysis**

**QacR_C72A_C141S**

MWobs 23272, MWcalc 23271

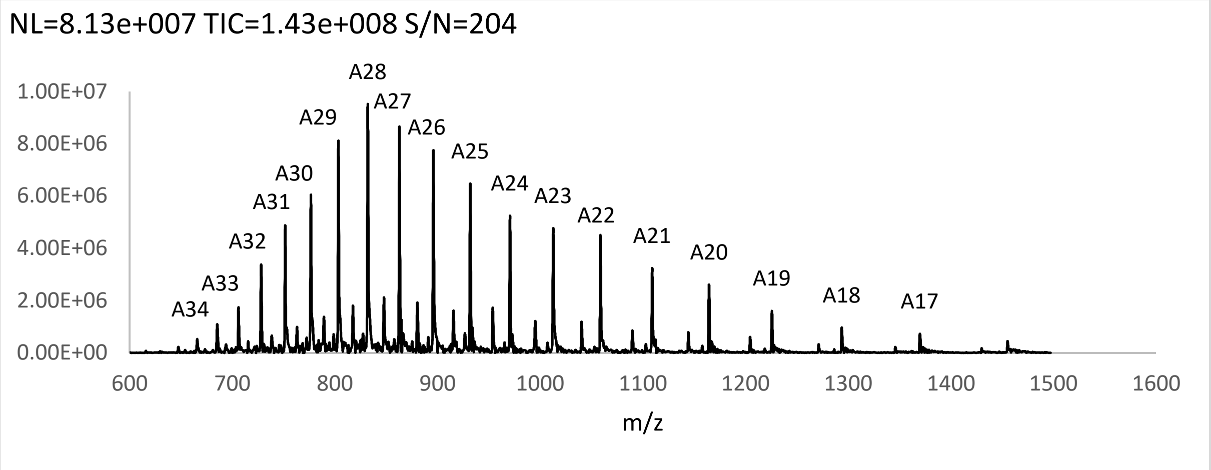


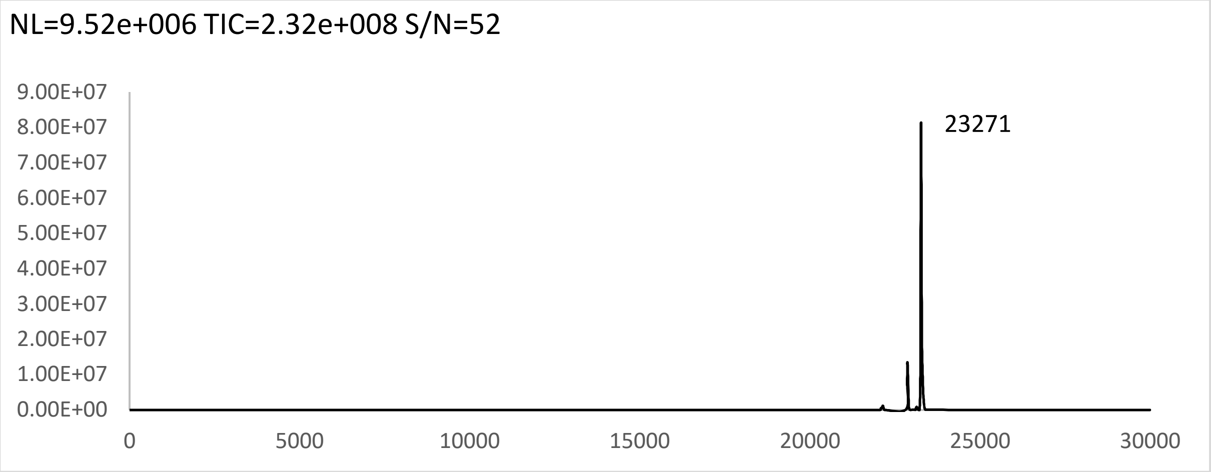


**RamR**

MWobs 23036, MWcalc23035 (without first methionine)

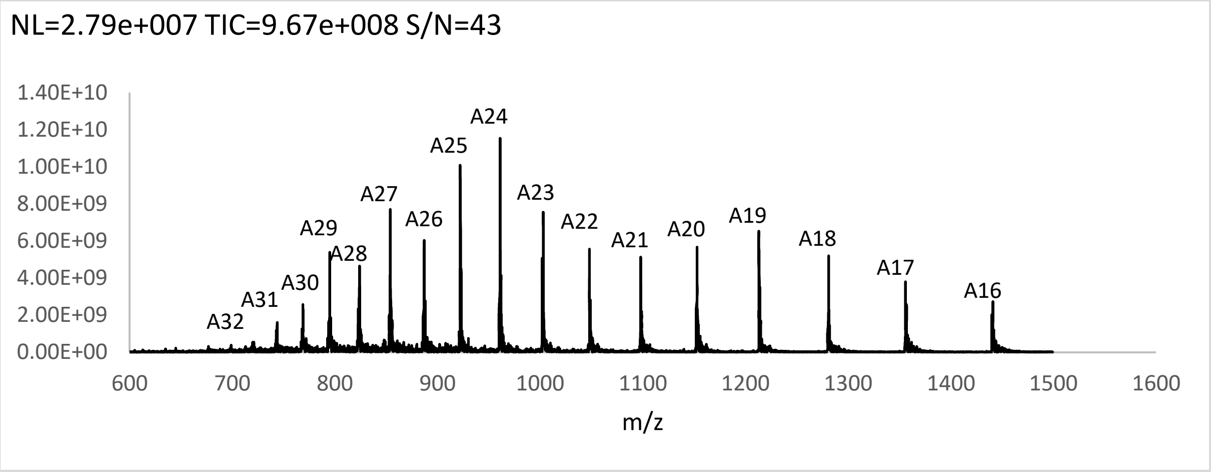

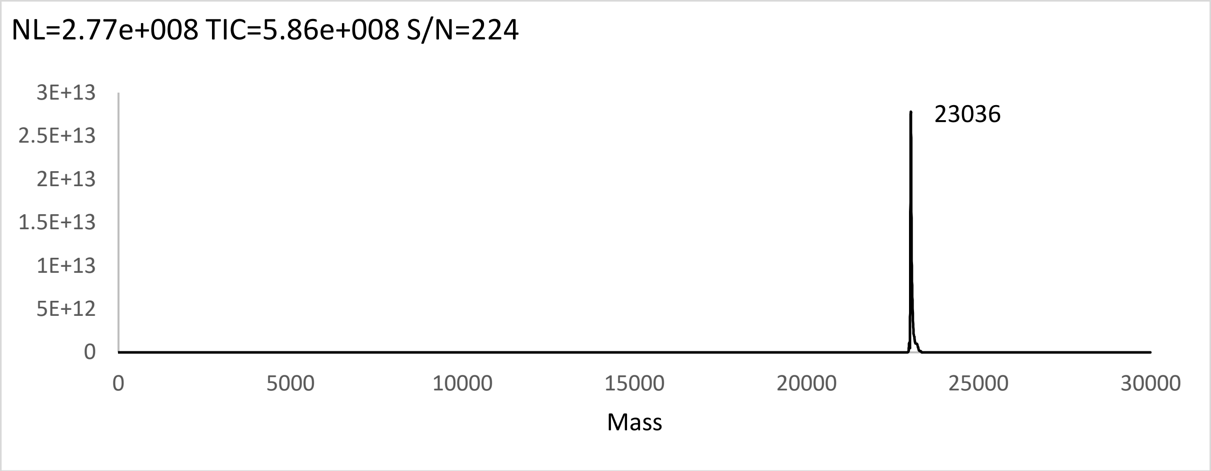


**CgmR**

MWobs 21454, MWcalc 21456

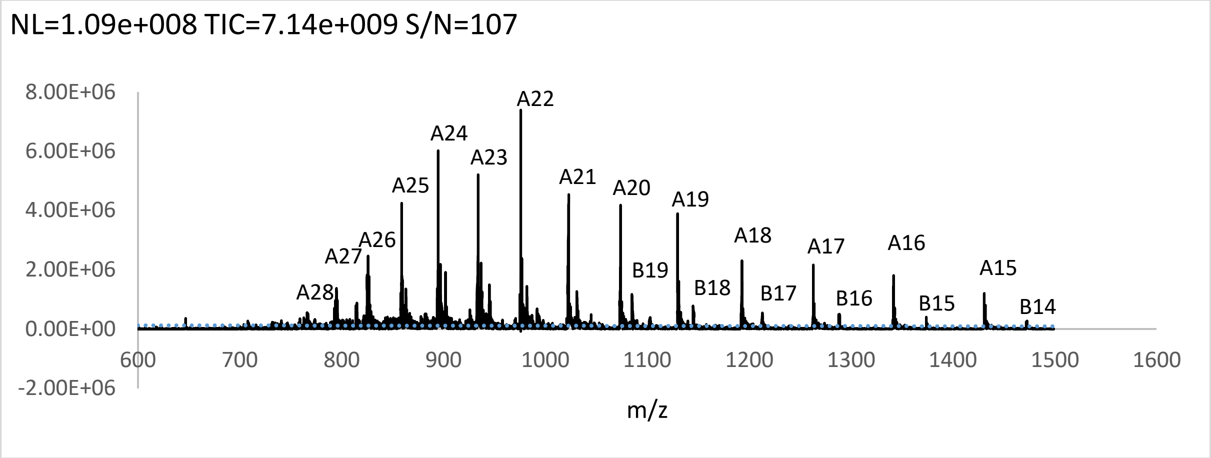


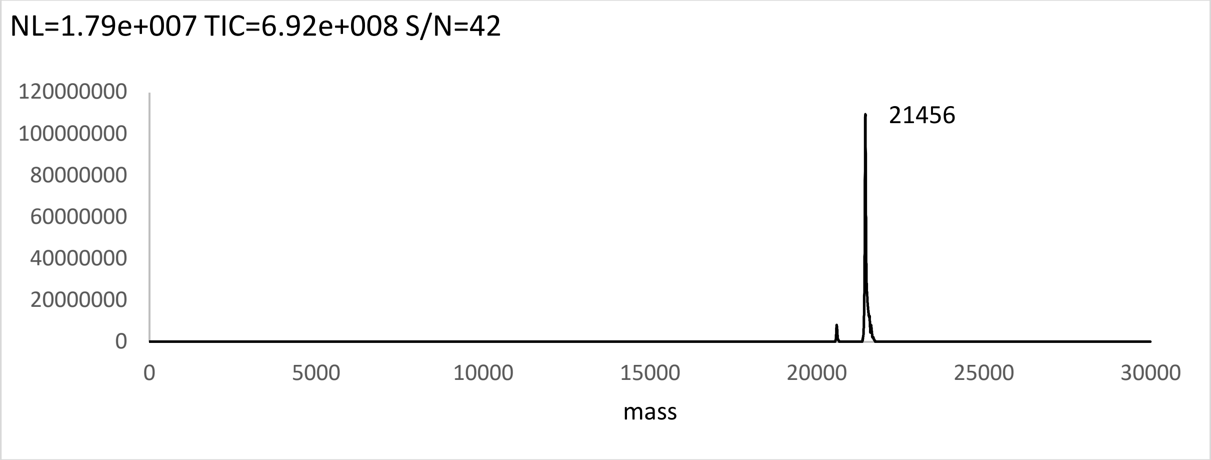

**Figure S4. Size exclusion chromatography**

**QacR_C72A_C141S**

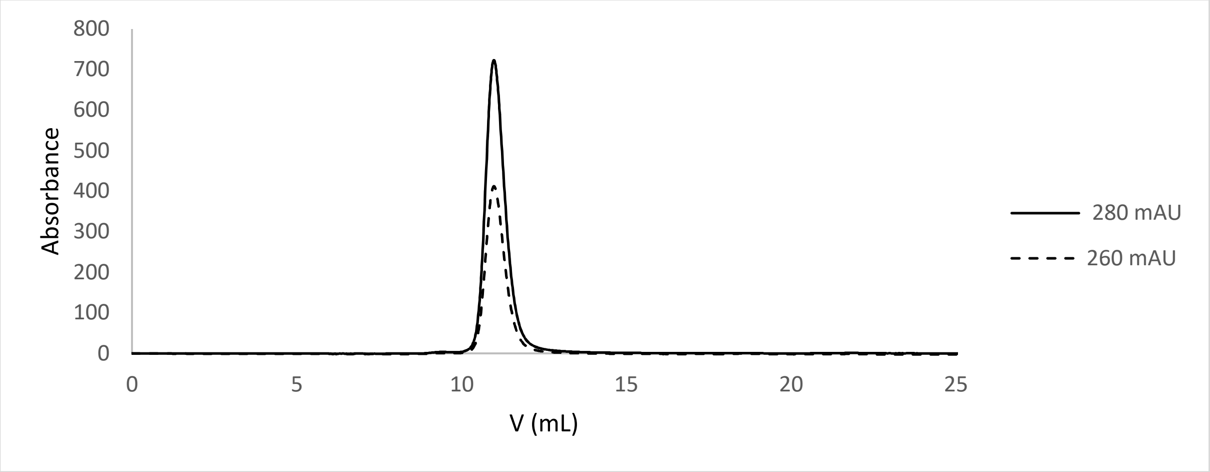


**RamR**

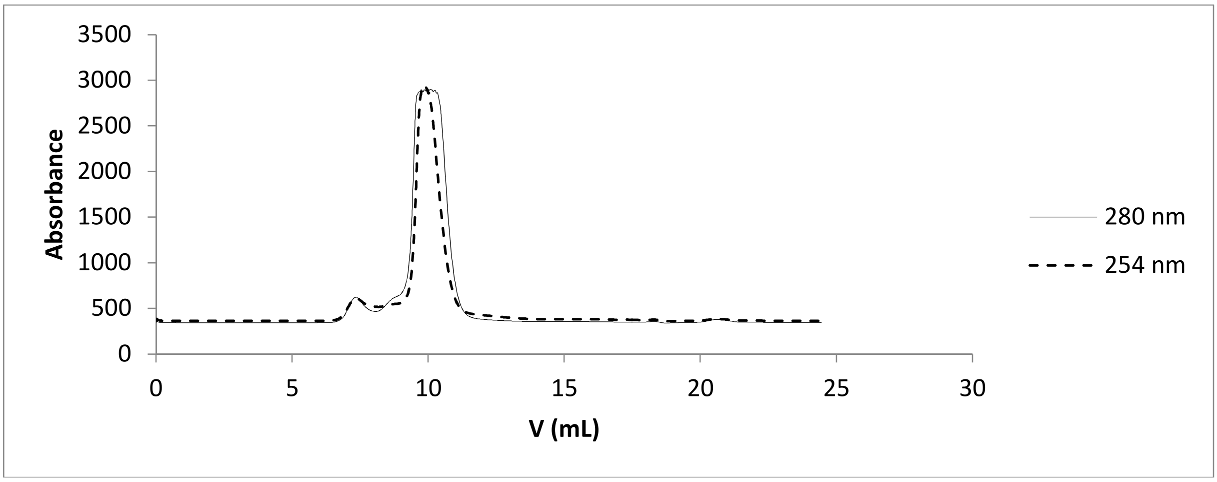


**CgmR**

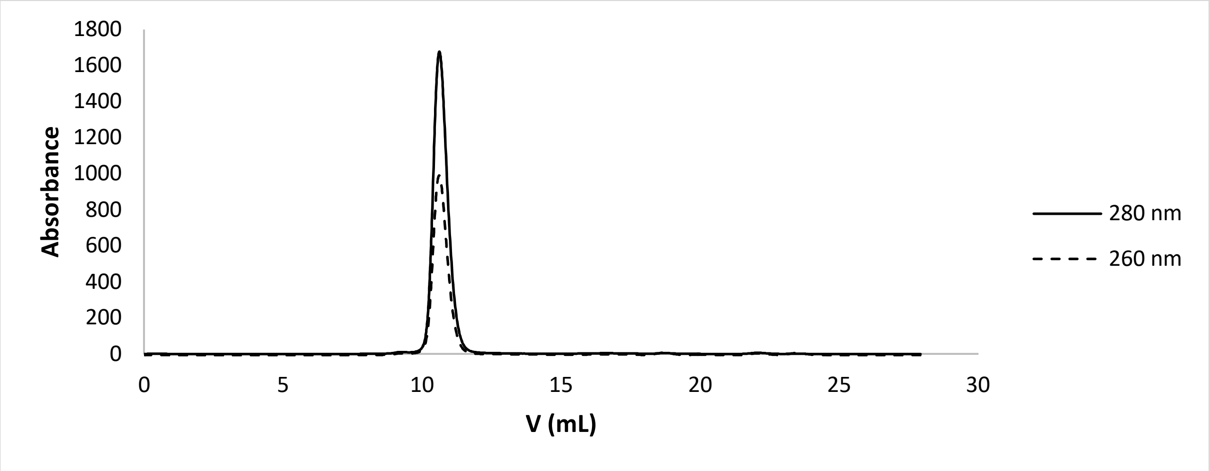

## 5. UV-visible titrations

UV-visible titrations were performed with 4 µM solution of the dimeric proteins titrated with 400 µM solution of $Cu(NO_3)_2$ in 20 mM MOPS, pH 7.0 containing 500 µM NaCl. The experiments with $Cu(phen)(NO_3)_2$ were performed with 80 µM solution of $Cu(phen)(NO_3)_2$ titrated with 400µM solution of the dimeric proteins.

### Figure S5. UV-visible titrations

**QacR (C72A, C141S) 4 µM : x equivalents $Cu(NO_3)_2$**

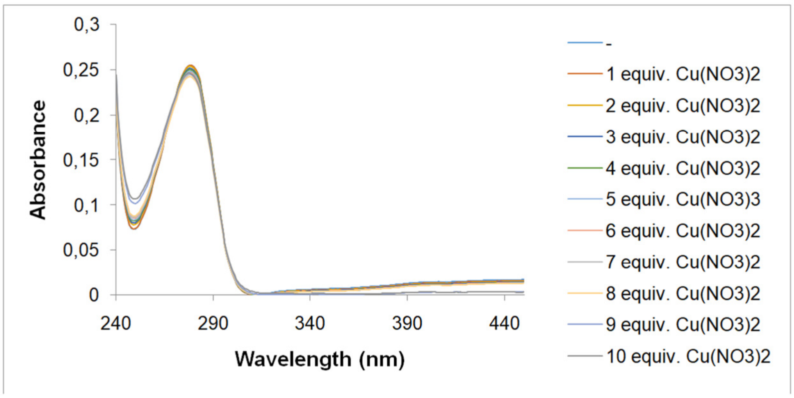


**80 µM $Cu(phen)(NO_3)_2$ : x equivalents QacR (C72A, C141S)**

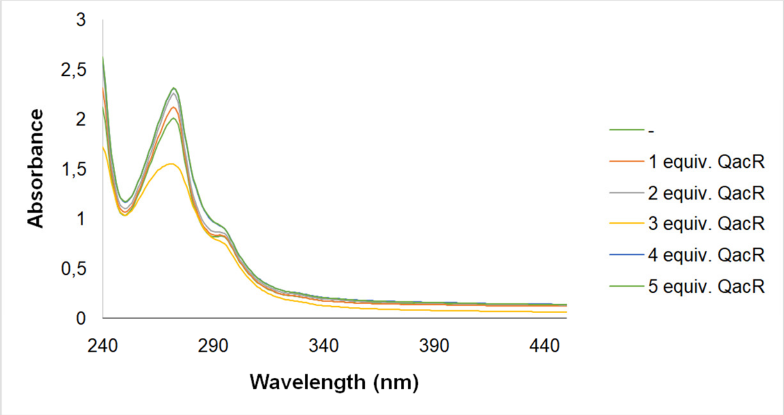


**RamR 4 µM : x equivalents $Cu(NO_3)_2$**

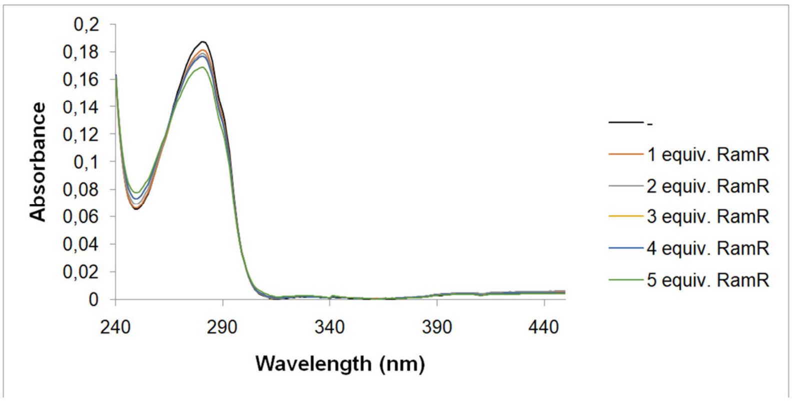

**80 µM $Cu(phen)(NO_3)_2$ : x equivalents RamR**

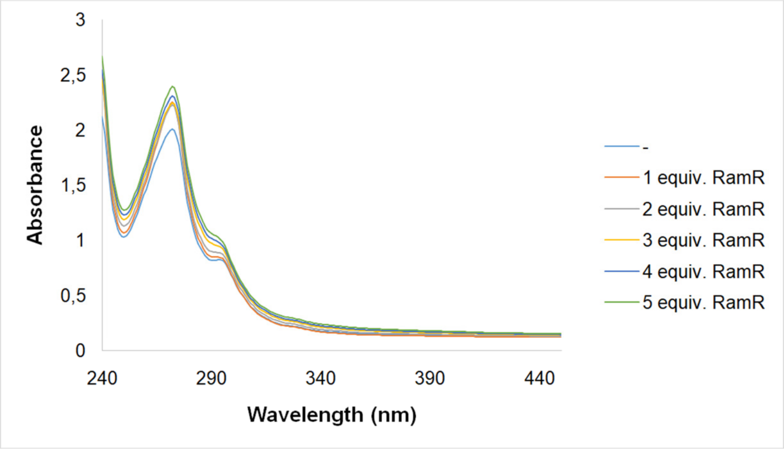


**CgmR 4 µM : x equivalents $Cu(NO_3)_2$**

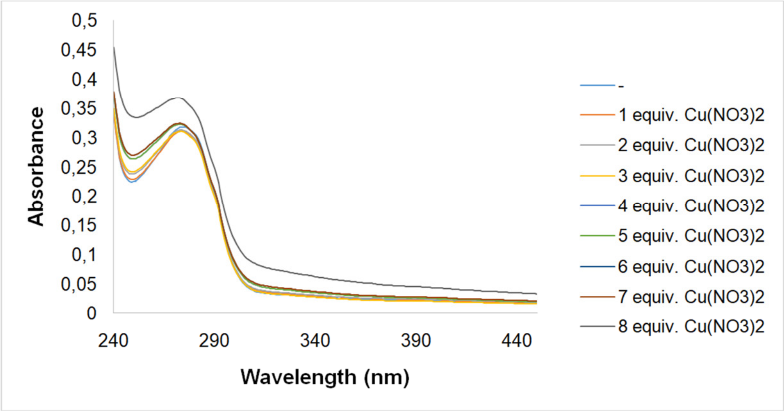


**CgmR : $Cu(phen)(NO_3)_2$**

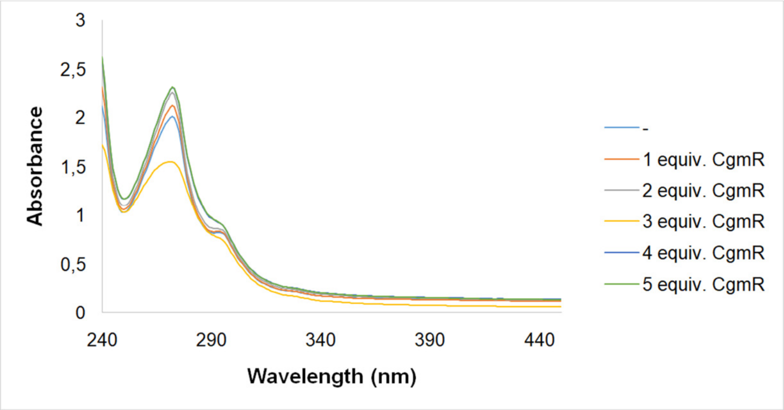

## 6. Catalysis: Friedel-Crafts alkylation

Catalytic reactions were performed in 150 µL total volume containing 90 µM $Cu(NO_3)_2$ (9 mol%) and 120 µM proteins (monomer, 1.3 equivalents), 1 mM of substrate **1** and **2** in 20 mM MOPS buffer pH 7.0 (or 20 mM MES buffer pH 5.0), 500 m M NaCl. In a typical procedure, the protein and the metal solutions were incubated for an hour under continuous inversion at 4 °C prior to addition of the substrates. Reactions were then incubated under continuous inversion at 4 °C for 72 h after which 50 µL of a 1 mM solution of 2-phenylquinoline in 20 mM MOPS buffer pH 7.0, 500 mM NaCl, 20% $CH_3CN$ were added. Reactions were extracted 3 times with 600 µL diethylether and the organic layers were dried over $Na_2SO_4$ and evaporated *in vacuo*. The resulting products were re-dissolved in 150 µL heptane:isopropanol 9:1 and analyzed by chiral HPLC (Chiralpak AD). Yields of all the catalytic reactions are based on peak areas at 275 nm using 2-phenylquinoline as internal standard.

**Table S1. Enone and indole scope of Friedel-Crafts reactions catalyzed by QacR, RamR and CgmR-based artificial metalloenzymes**

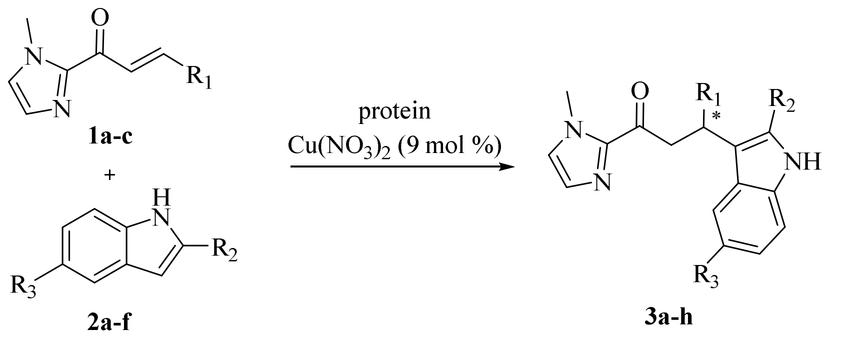


**1a** R1: methyl
**1b** R1: phenyl
**1c** R1: *tert*-butyl
**2a** R2: methyl; R3 H
**2b** R2: H; R3: H
**2c** R2: phenyl; R3: H
**2d** R2: H; R3: methoxyl
**2e** R2: H; R3: chlorine
**2f** R2: H; R3: bromine

| Entry | catalyst | enone | indole | product | Yield (%) | ee (%) |
|---|---|---|---|---|---|---|
| 1 | $Cu^{2+}$ | **1a** | **2a** | **3a** | 22 ± 7 | - |
| 2 | $Cu^{2+}$⊂RamR | **1a** | **2a** | **3a** | 57 ± 9 | 29 ± 3 (-) |
| 3 | $Cu^{2+}$⊂CgmR | **1a** | **2a** | **3a** | 52 ± 12 | 13 ± 3 (-) |
| 4 | $Cu^{2+}$⊂QacR | **1a** | **2a** | **3a** | 78 ± 11 | 34 ± 3 (-) |
| 5 | $Cu^{2+}$ | **1a** | **2b** | **3b** | 7 ± 8 | - |
| 6 | $Cu^{2+}$⊂RamR | **1a** | **2b** | **3b** | 5 ± 5 | n.d. |
| 7 | $Cu^{2+}$⊂CgmR | **1a** | **2b** | **3b** | 7 ± 3 | 27 ± 3 (+)-*R* |
| 8 | QacR⊂$Cu^{2+}$ | **1a** | **2b** | **3b** | 27 ± 12 | 26 ± 2 (+)-*R* |
| 9 | $Cu^{2+}$ | **1a** | **2c** | **3c** | < 5 | - |
| 10 | $Cu^{2+}$⊂RamR | **1a** | **2c** | **3c** | < 5 | n.d. |
| 11 | $Cu^{2+}$⊂CgmR | **1a** | **2c** | **3c** | < 5 | n.d. |
| 12 | $Cu^{2+}$⊂QacR | **1a** | **2c** | **3c** | < 5 | n.d. |
| 13 | $Cu^{2+}$ | **1a** | **2d** | **3d** | 10 ± 6 | - |
| 14 | $Cu^{2+}$⊂RamR | **1a** | **2d** | **3d** | 19 ± 3 | 35 ± 3 (+) |
| 15 | $Cu^{2+}$⊂CgmR | **1a** | **2d** | **3d** | 31 ± 14 | 37 ± 1 (+) |
| 16 | $Cu^{2+}$⊂QacR | **1a** | **2d** | **3d** | 39 ± 10 | 9 ± 4 (+) |
| 17 | $Cu^{2+}$ | **1a** | **2e** | **3e** | Y < 5 | - |
| 18 | $Cu^{2+}$⊂RamR | **1a** | **2e** | **3e** | Y < 5 | n.d. |
| 19 | $Cu^{2+}$⊂CgmR | **1a** | **2e** | **3e** | Y < 5 | n.d. |
| 20 | $Cu^{2+}$⊂QacR | **1a** | **2e** | **3e** | 17 ± 8 | 38 ± 5 (+) |
| 21 | $Cu^{2+}$ | **1a** | **2f** | **3f** | < 5 | - |
| 22 | $Cu^{2+}$⊂RamR | **1a** | **2f** | **3f** | < 5 | n.d. |
| 23 | $Cu^{2+}$⊂CgmR | **1a** | **2f** | **3f** | < 5 | n.d. |
| 24 | $Cu^{2+}$⊂QacR | **1a** | **2f** | **3f** | < 5 | n.d. |
| 25 | $Cu^{2+}$ | **1b** | **2b** | **3g** | 54 ± 9 | - |
| 26 | $Cu^{2+}$⊂RamR | **1b** | **2b** | **3g** | < 5 | n.d. |
| 27 | $Cu^{2+}$⊂CgmR | **1b** | **2b** | **3g** | 18 ± 4 | 6 ± 2 |
| 28 | $Cu^{2+}$⊂QacR | **1b** | **2b** | **3g** | 59 ± 7 | 75 ± 4 |
| 29 | $Cu^{2+}$ | **1c** | **2b** | **3h** | < 5 | - |
| 30 | $Cu^{2+}$⊂RamR | **1c** | **2b** | **3h** | < 5 | n.d. |
| 31 | $Cu^{2+}$⊂CgmR | **1c** | **2b** | **3h** | < 5 | n.d. |
| 32 | $Cu^{2+}$⊂QacR | **1c** | **2b** | **3h** | < 5 | n.d. |

Typical conditions: 90 µM $Cu(NO_3)_2$ (9 mol%) loading with 1.3 eq of protein (120 µM), 1 mM of substrate **1** and **2** in 20 mM MOPS buffer pH 7.0, 500 mM NaCl, for 72 h at 4 °C. All the results listed correspond to the average of two independent experiments, each carried out in duplicate. Errors listed are standard deviations.[a] Yields were determined by HPLC and using 2-phenylquinoline as internal standard. [b] Sign of rotation was assigned by comparison with elution order in chiral HPLC.[3],[6] For yields <5% ee's were not determined.

**Table S2. Control experiments for catalytic vinylogous Friedel–Crafts alkylation reactions**

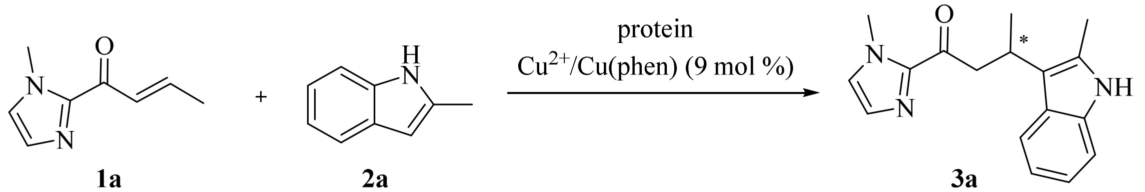


| Entry | catalyst | Yield (%) | ee (%) |
|---|---|---|---|
| 1 | - | < 5 | n.d. |
| 2 | $Cu^{2+}$ | 22 ± 7 | - |
| 3 | Cu(phen) | 43 ± 2 | 7 ± 3 (-) |
| 4 | RamR | 5 ± 6 | 8 ± 6 (-) |
| 5 | $Cu^{2+}$⊂RamR | 57 ± 9 | 29 ± 3 (-) |
| 6 | Cu(phen) ⊂RamR | 21 ± 13 | 34 ± 7 (-) |
| 7 | CgmR | 6 ± 3 | 10 ± 21 (-) |
| 8 | $Cu^{2+}$⊂CgmR | 52 ± 12 | 13 ± 3 (-) |
| 9 | Cu(phen) ⊂CgmR | 30 ± 3 | 15 ± 1 (-) |
| 10 | QacR | 11 ± 7 | 13 ± 19 (-) |
| 11 | $Cu^{2+}$⊂QacR | 78 ± 11 | 34 ± 3 (-) |
| 12 | Cu(phen) ⊂QacR | 36 ± 6 | 30 ± 2 (-) |

Typical conditions: 90 µM $Cu(NO_3)_2$ (9 mol %) loading with 1.3 eq of protein (120 µM), 1 mM of substrate **1** and **2** in 20 mM MOPS buffer pH 7.0, 500 mM NaCl, for 72 h at 4 °C. All the results listed correspond to the average of two independent experiments, each carried out in duplicate. Errors listed are standard deviations.[a] Yields were determined by HPLC and using 2-phenylquinoline as internal standard. [b] Sign of rotation was assigned by elution order in chiral HPLC. For yields <5% ee's were not determined.

**Table S3. Inhibition experiments for catalytic vinylogous Friedel–Crafts alkylation reactions**

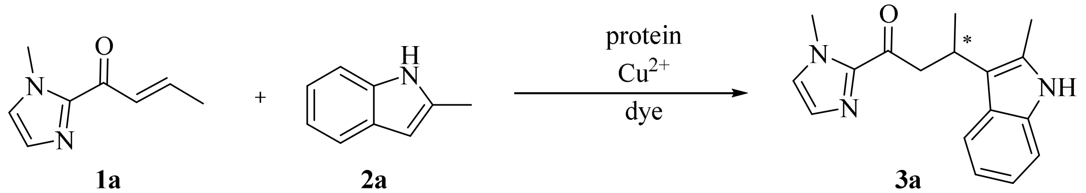


| Entry | Catalyst | Dye/equivalents | Yield (%) | ee (%) |
|---|---|---|---|---|
| 1 | $Cu(NO_3)_2$ | - | 22 ± 7 | - |
| 2[a] | $Cu(NO_3)_2$ | Ethidium/1 | 13 ± 0 | - |
| 3[a] | $Cu(NO_3)_2$ | Ethidium/4 | 13 ± 1 | - |
| 4[a] | $Cu(NO_3)_2$ | Ethidium/8 | 12 ± 0 | - |
| 5[a] | $Cu(NO_3)_2$ | R6G/1 | 12 ± 1 | - |
| 6[a] | $Cu(NO_3)_2$ | R6G/4 | 10 ± 1 | - |
| 7[a] | $Cu(NO_3)_2$ | R6G/8 | 12 ± 1 | - |
| 8 | $Cu^{2+}$⊂QacR | - | 78 ± 11 | 34 ± 3 (-) |
| 9 | $Cu^{2+}$⊂QacR | Ethidium/1 | 60 ± 7 | 25 ± 1 (-) |
| 10 | $Cu^{2+}$⊂QacR | Ethidium/4 | 36 ± 9 | 5 ± 3 (+) |
| 11 | $Cu^{2+}$⊂QacR | Ethidium/8 | 27 ± 10 | 9 ± 2 (+) |
| 12 | $Cu^{2+}$⊂QacR | R6G/1 | 76 ± 9 | 32 ± 1 (-) |
| 13 | $Cu^{2+}$⊂QacR | R6G/4 | 44 ± 4 | 15 ± 4 (-) |
| 14 | $Cu^{2+}$⊂QacR | R6G/8 | 33 ± 17 | 7 ± 9 (-) |
| 15 | $Cu^{2+}$⊂RamR | - | 57 ± 9 | 29 ± 3 (-) |
| 16 | $Cu^{2+}$⊂RamR | Ethidium/1 | 32 ± 8 | 32 ± 4 (-) |
| 17 | $Cu^{2+}$⊂RamR | Ethidium/4 | 26 ± 6 | 31 ± 0 (-) |
| 18 | $Cu^{2+}$⊂RamR | Ethidium/8 | 17 ± 3 | 29 ± 2 (-) |
| 19 | $Cu^{2+}$⊂CgmR | - | 52 ± 12 | 13 ± 3 (-) |
| 20 | $Cu^{2+}$⊂CgmR | Ethidium/1 | 39 ± 11 | 3 ± 2 (-) |
| 21 | $Cu^{2+}$⊂CgmR | Ethidium/4 | 25 ± 9 | 6 ± 1 (+) |
| 22 | $Cu^{2+}$⊂CgmR | Ethidium/8 | 18 ± 4 | 11 ± 1 (+) |

[a]The reactions were performed in duplicate.

**Table S4. Control Friedel-Crafts reaction with dialyzed $Cu^{2+}$⊂protein**

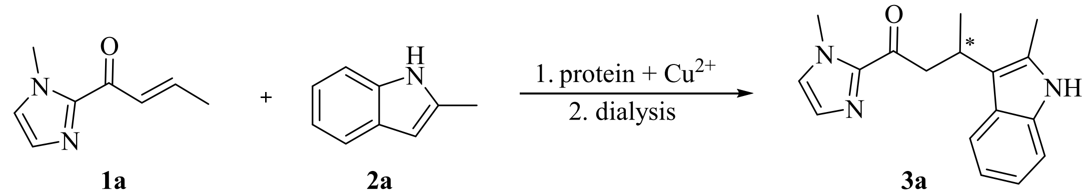


**1a** **2a** **3a**

⊂

| Entry | Protein | Dialysis | Yield (%) | ee (%) |
|---|---|---|---|---|
| 1 | $Cu^{2+}$⊂QacR | NO | 78 ± 11 | 34 ± 3 |
| 2 | $Cu^{2+}$⊂QacR | YES | 29 ± 1 | 48 ± 0 |
| 3 | $Cu^{2+}$⊂RamR | NO | 57 ± 9 | 29 ± 3 |
| 4 | $Cu^{2+}$⊂RamR | YES | 13 ± 2 | 43 ± 4 |
| 5 | $Cu^{2+}$⊂CgmR | NO | 52 ± 12 | 13 ± 3 |
| 6 | $Cu^{2+}$⊂CgmR | YES | 24 ± 8 | 15 ± 2 |

Typical conditions: The proteins were incubated with 1.1 excess of $Cu(NO_3)_2$ for 1 h. Subsequently, the 1 mL samples were dialyzed against 1 L of 20 mM MOPS, 500 mM NaCl, pH7 (reaction buffer) overnight and the recovered samples were then used for catalysis as previously described. The reactions were performed in duplicates.

**Figure S6: calibration curve and chiral HPLC traces obtained for biocatalytic reactions with product 3a**

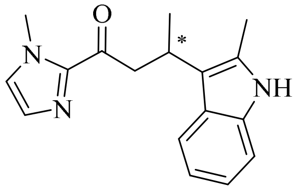

**3a**

**S6.1. Calibration curve**

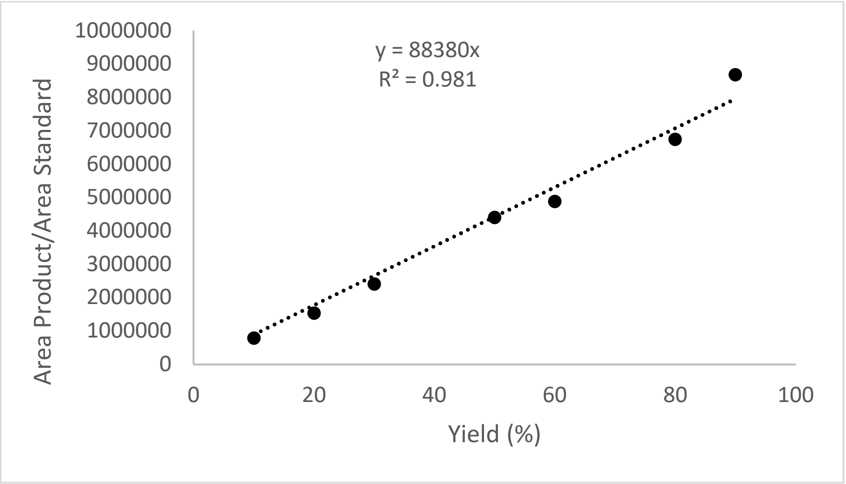

### S6.2. Catalyst: $Cu(NO_3)_2$

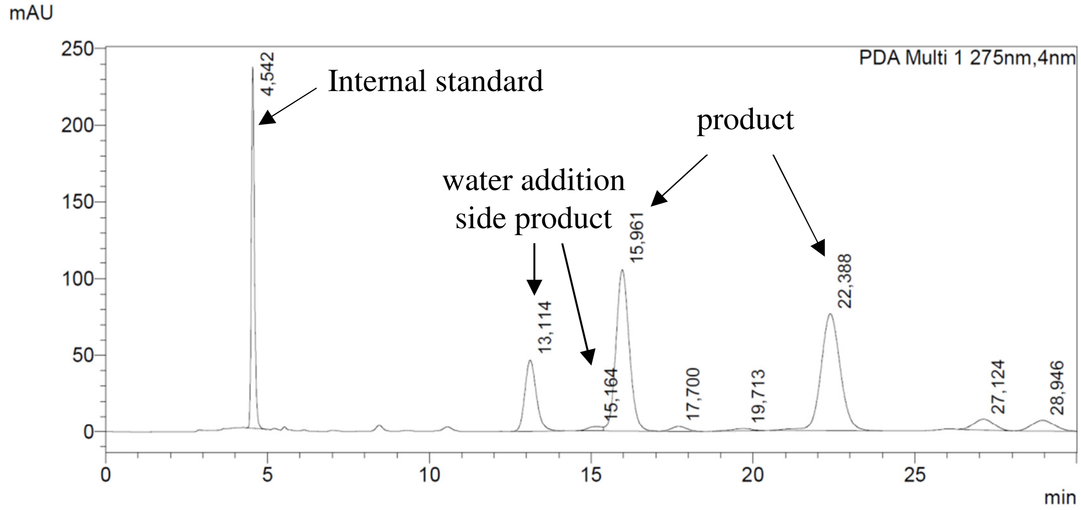


### S6.3. Catalyst: $Cu^{2+}$⊂RamR

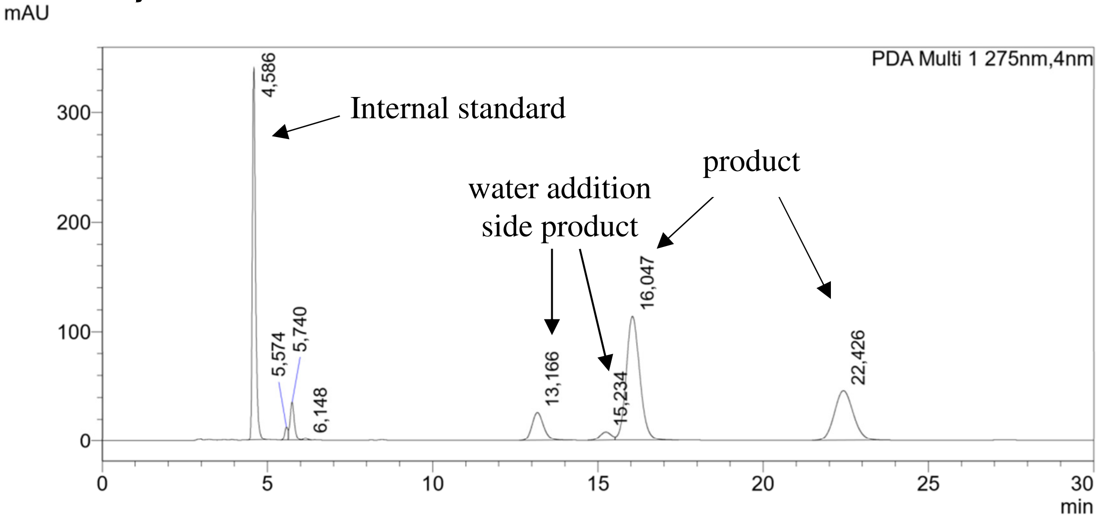


### S6.4. Catalyst: RamR

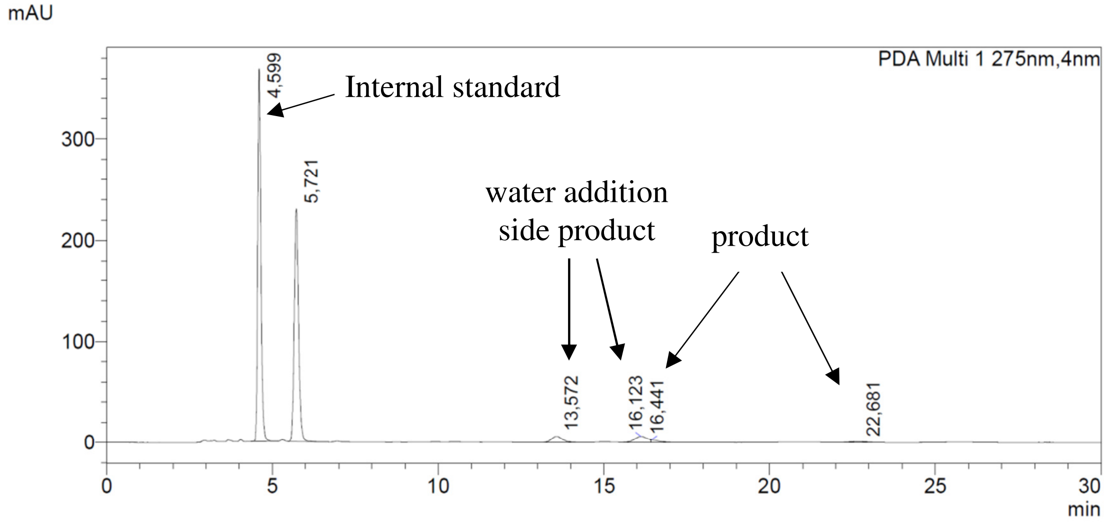

**S6.5. Catalyst: $Cu^{2+}$⊂CgmR**

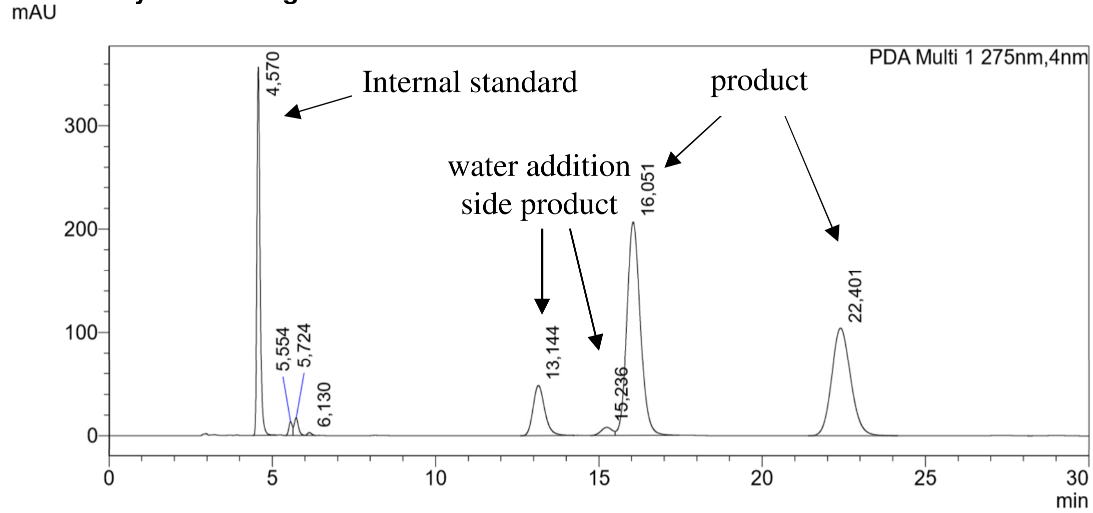


**S6.6. Catalyst: CgmR**

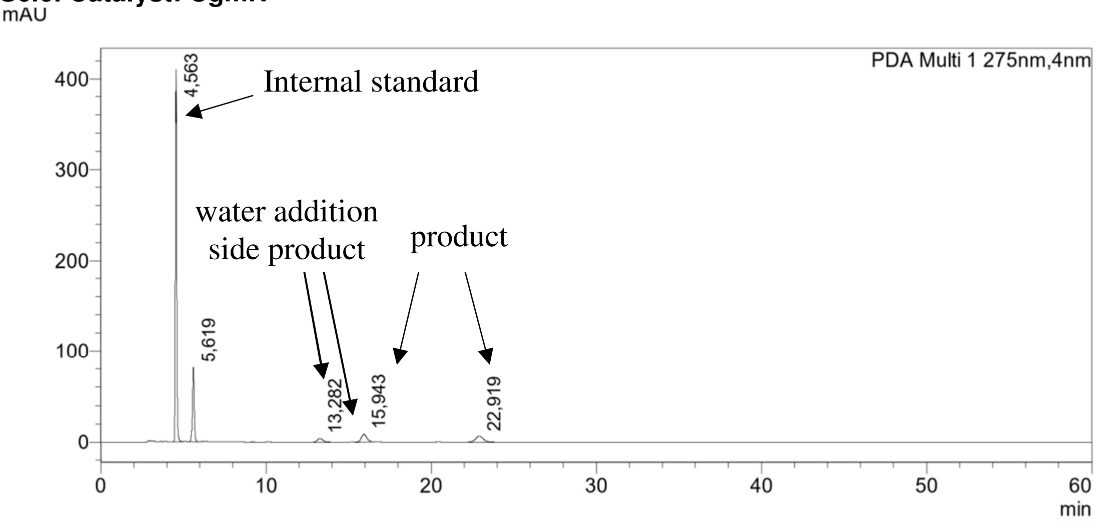


**S6.7. Catalyst: $Cu^{2+}$⊂QacR**

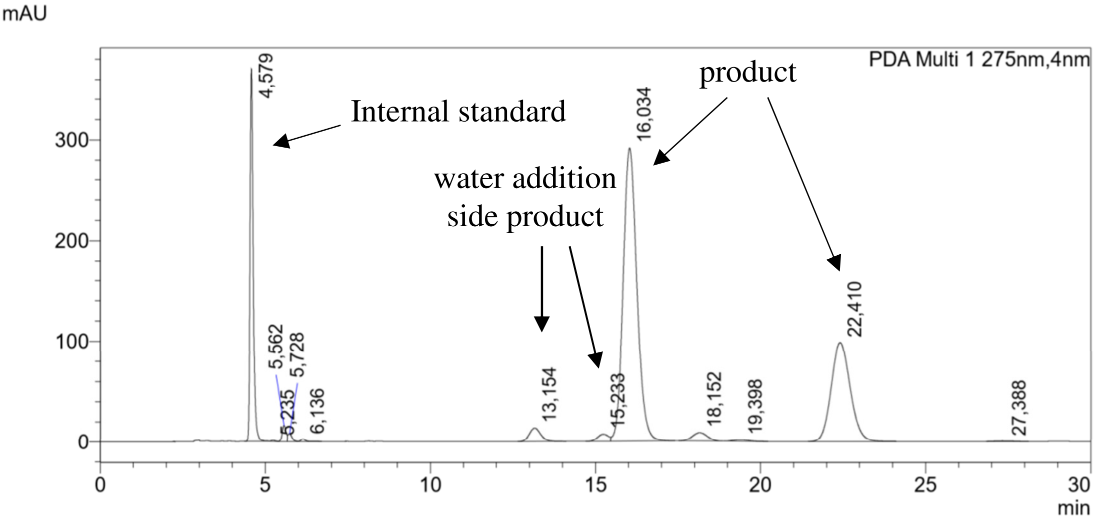

### S6.8. Catalyst: QacR

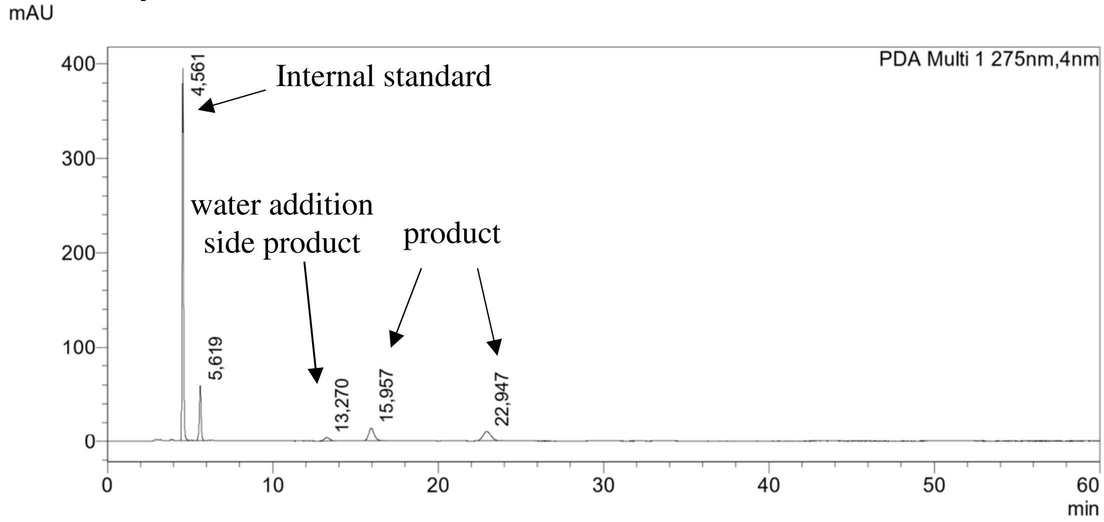


**Figure S7: calibration curve and chiral HPLC traces obtained for biocatalytic reactions with product 3b**

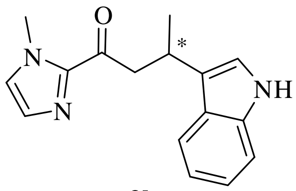


**3b**

### S7.1. Calibration curve

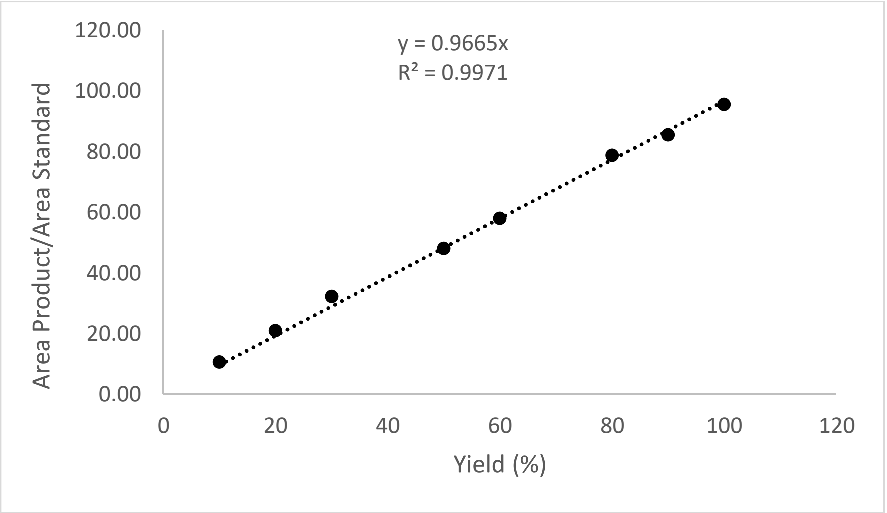

**S7.2. Catalyst: $Cu(NO_3)_2$**

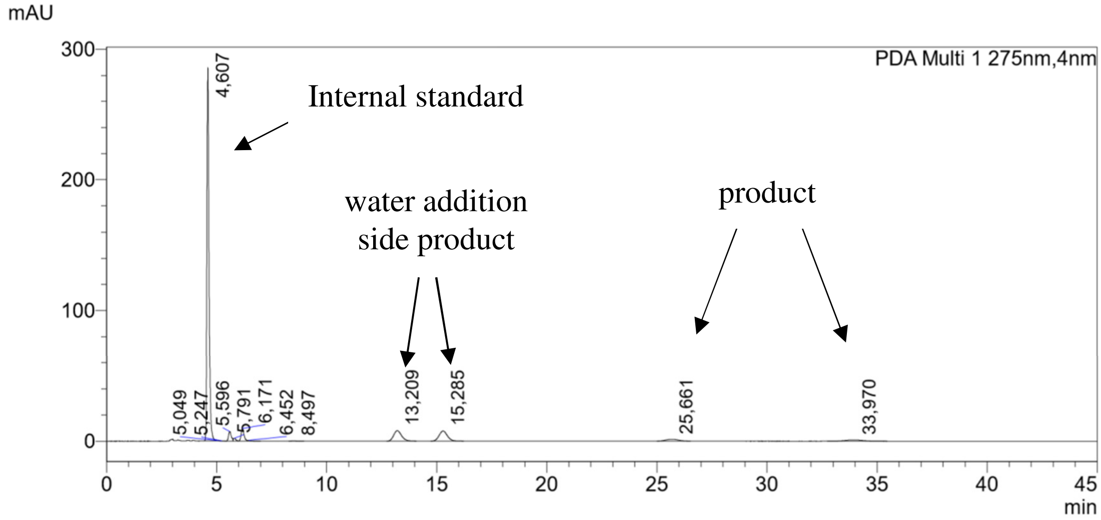


**S7.3. Catalyst: $Cu^{2+}$⊂RamR**

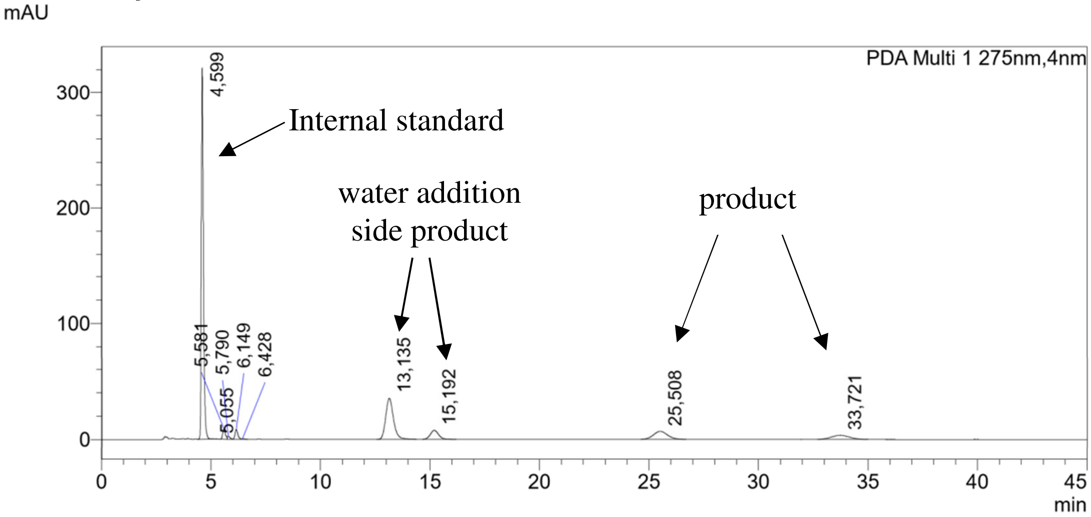


**S7.4. Catalyst: $Cu^{2+}$⊂CgmR**

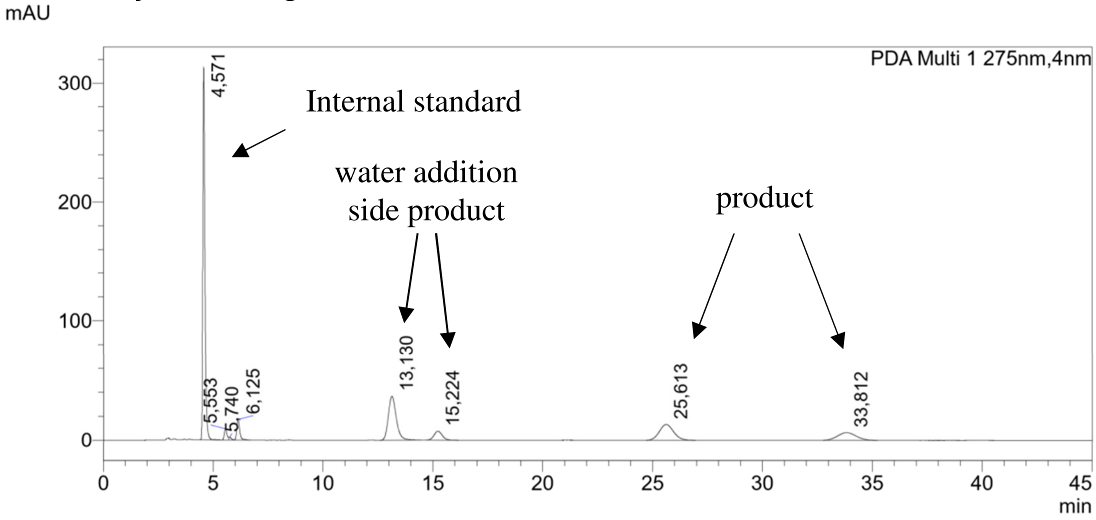

### S7.5. Catalyst: $Cu^{2+}$⊂QacR

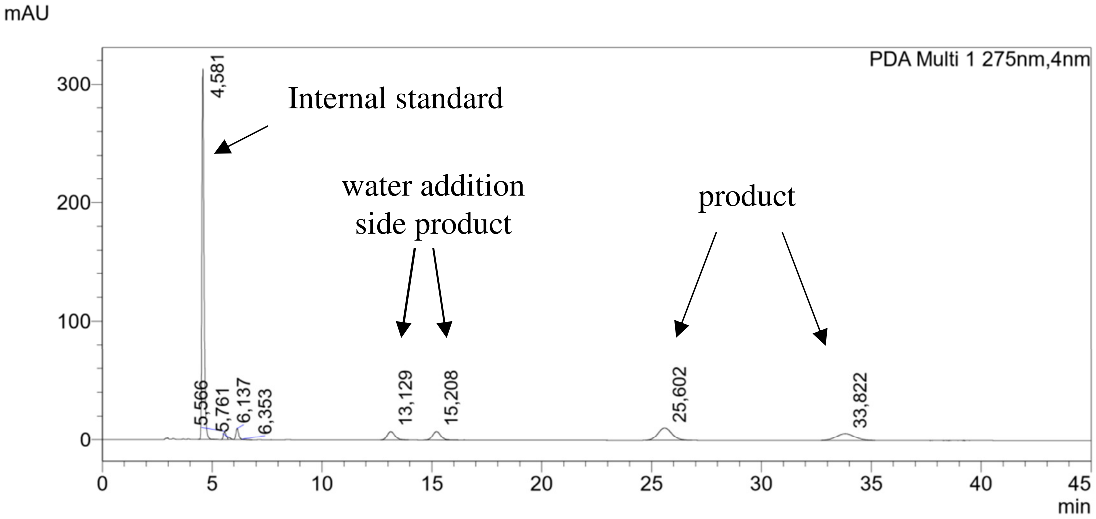


**Figure S8: calibration curve and chiral HPLC traces obtained for biocatalytic reactions with product 3c**

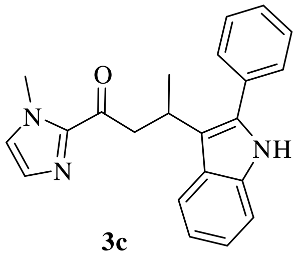


**3c**

### S8.1. Calibration curve

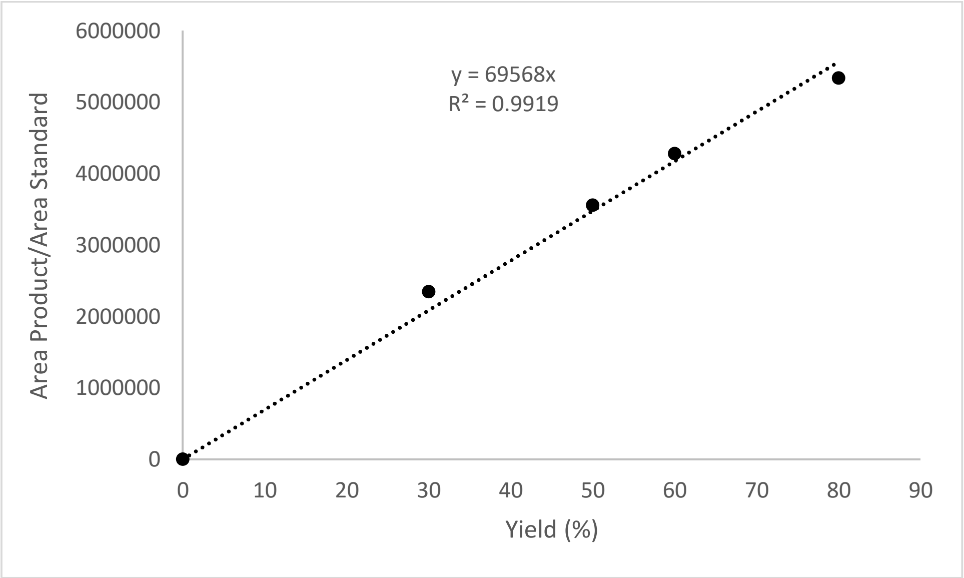

**S8.2. Catalyst: $Cu(NO_3)_2$**

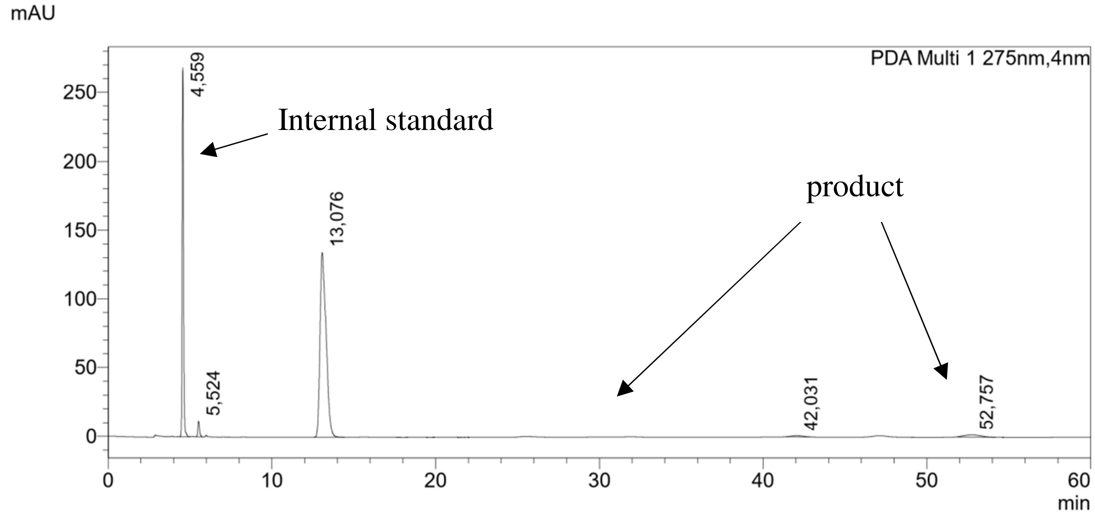


**S8.3. Catalyst: $Cu^{2+}$⊂RamR**

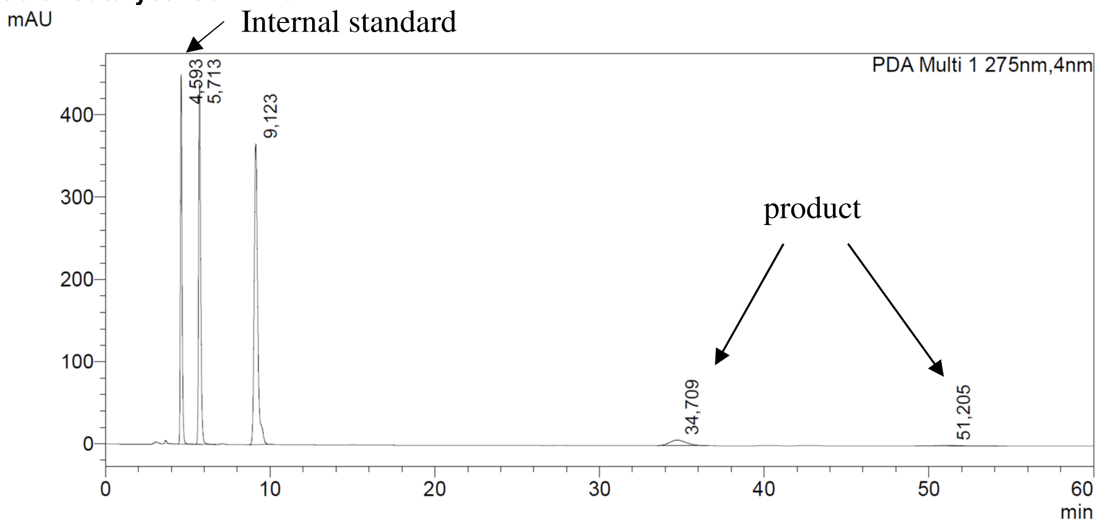


**S8.4. Catalyst: $Cu^{2+}$⊂CgmR**

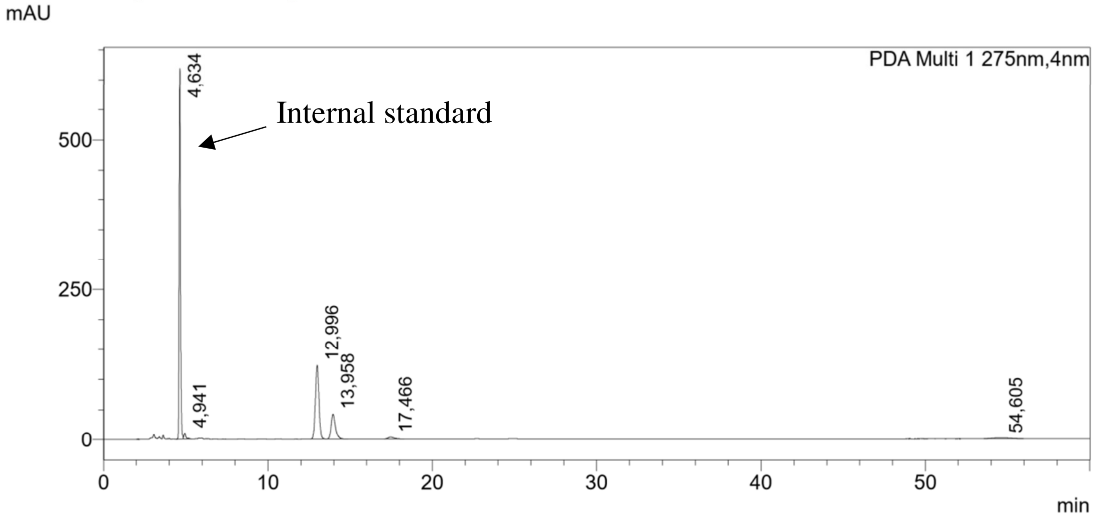

**Figure S9: calibration curve and chiral HPLC traces obtained for biocatalytic reactions with product 3d**

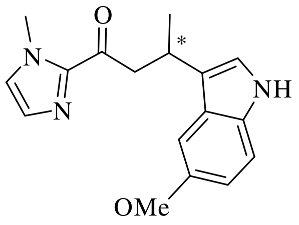


**3d**

**S9.1. Calibration curve**

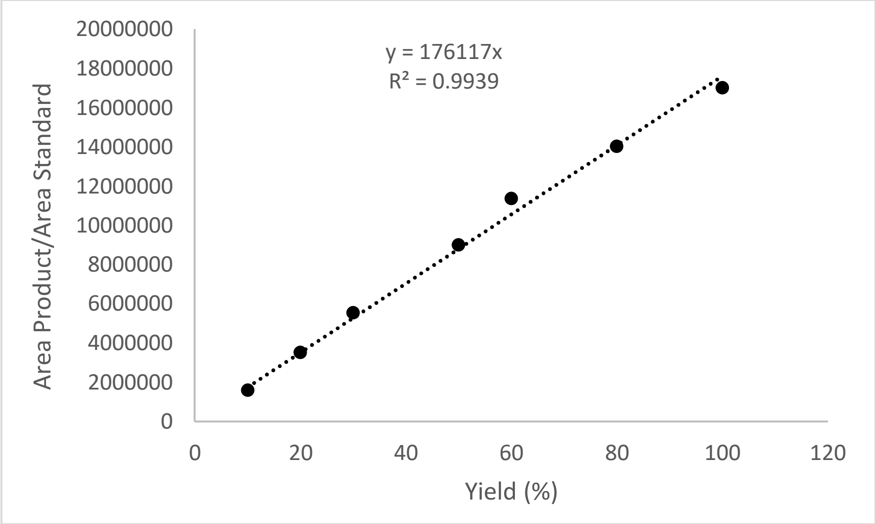


**S9.2. Catalyst: $Cu(NO_3)_2$**

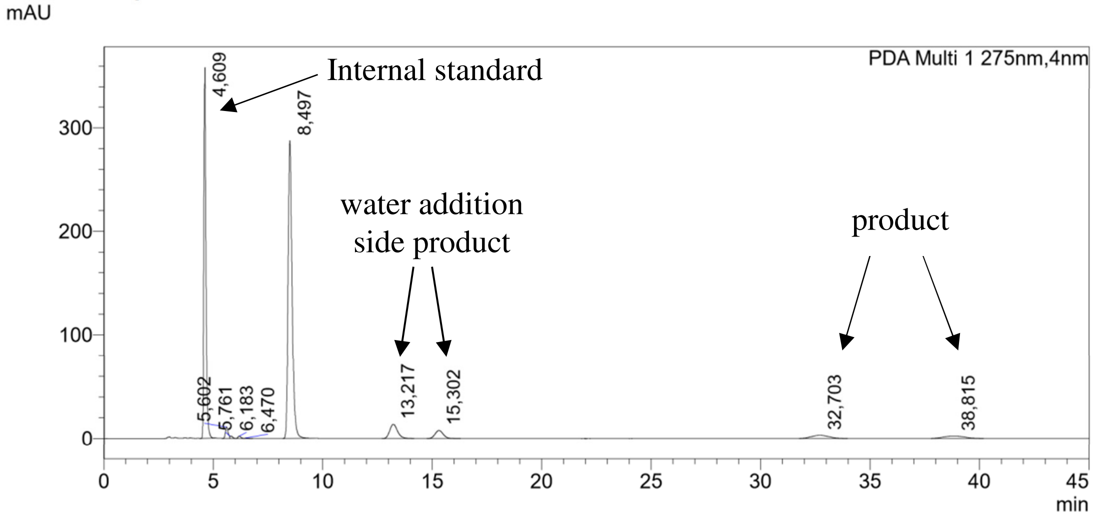

### S9.3. Catalyst: $Cu^{2+}$⊂RamR

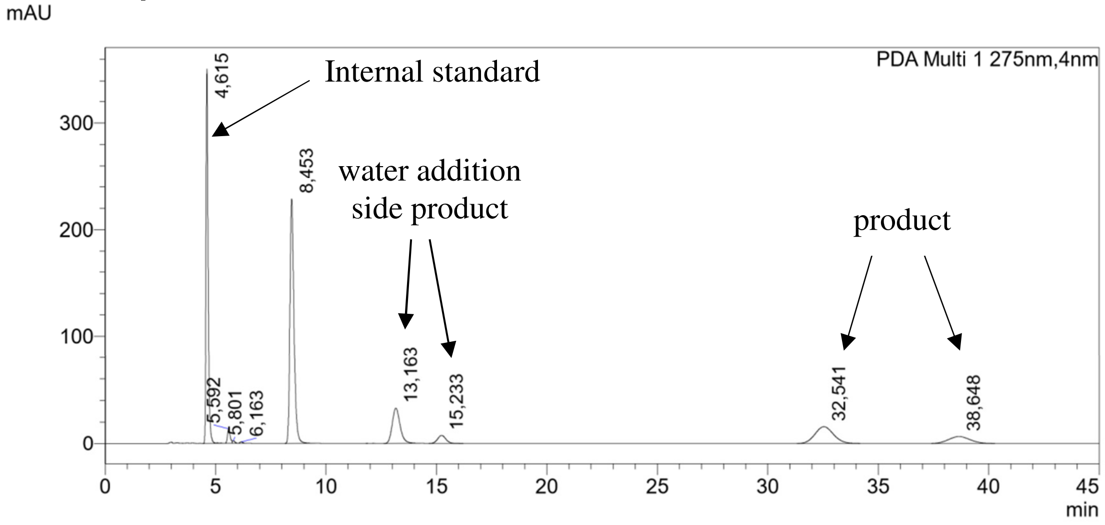


### S9.4. Catalyst: $Cu^{2+}$⊂CgmR

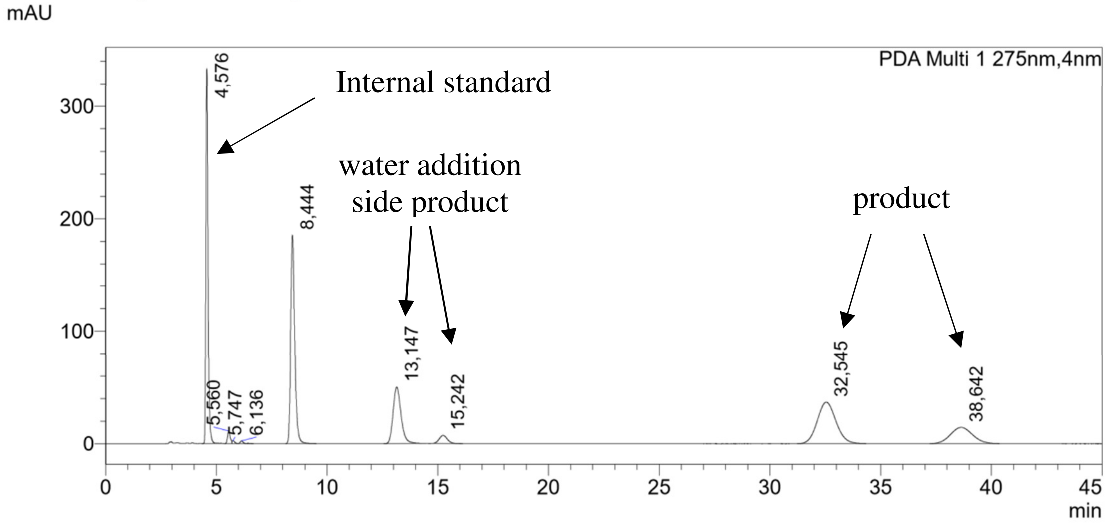


### S9.5. Catalyst: $Cu^{2+}$⊂QacR

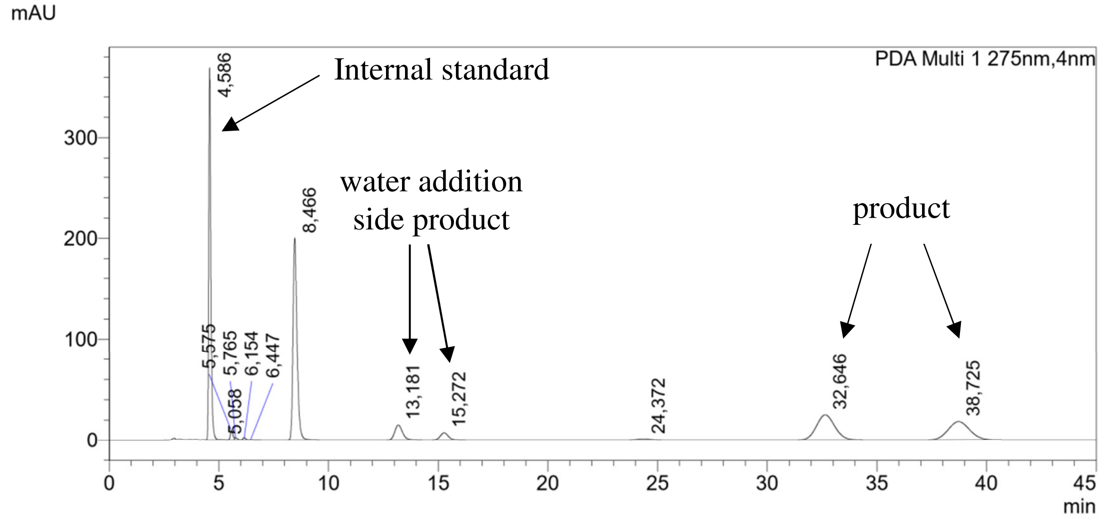

**Figure S10: calibration curve and chiral HPLC traces obtained for biocatalytic reactions with product 3e**

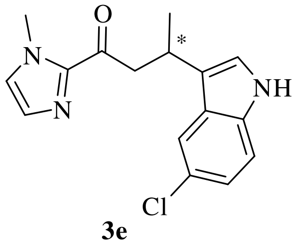


**3e**

### S10.1. Calibration curve

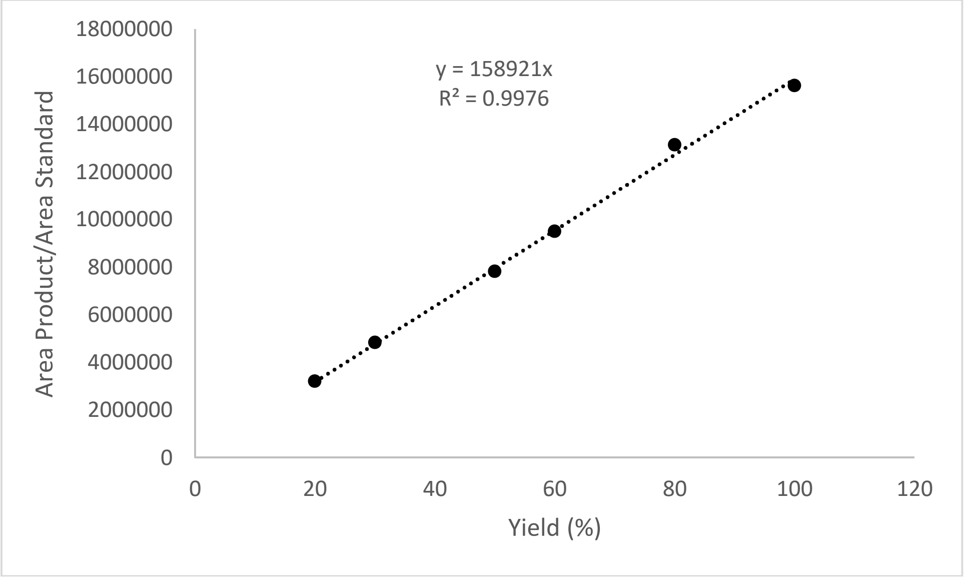


### S10.2. Catalyst: $Cu(NO_3)_2$

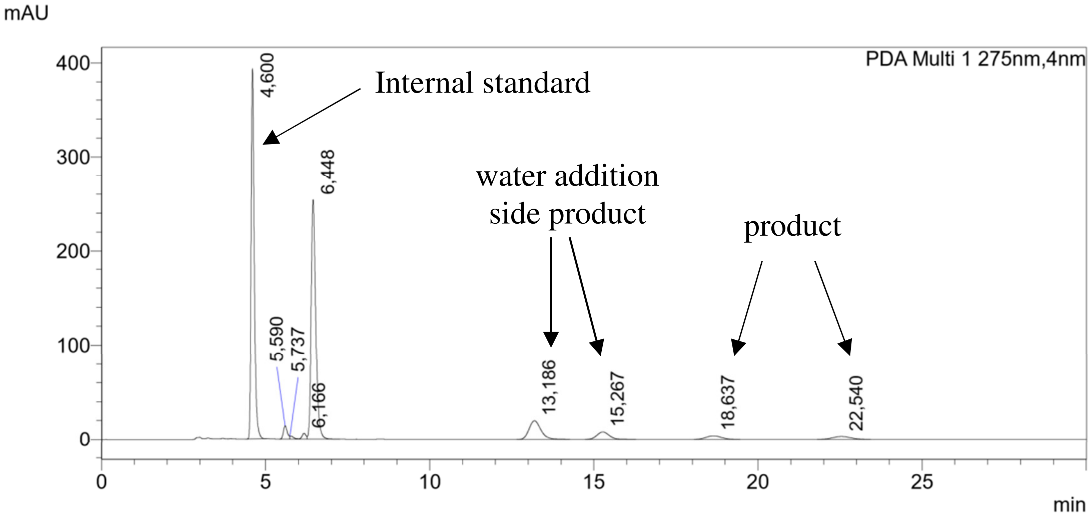

**S10.3. Catalyst: $Cu^{2+}$⊂RamR**

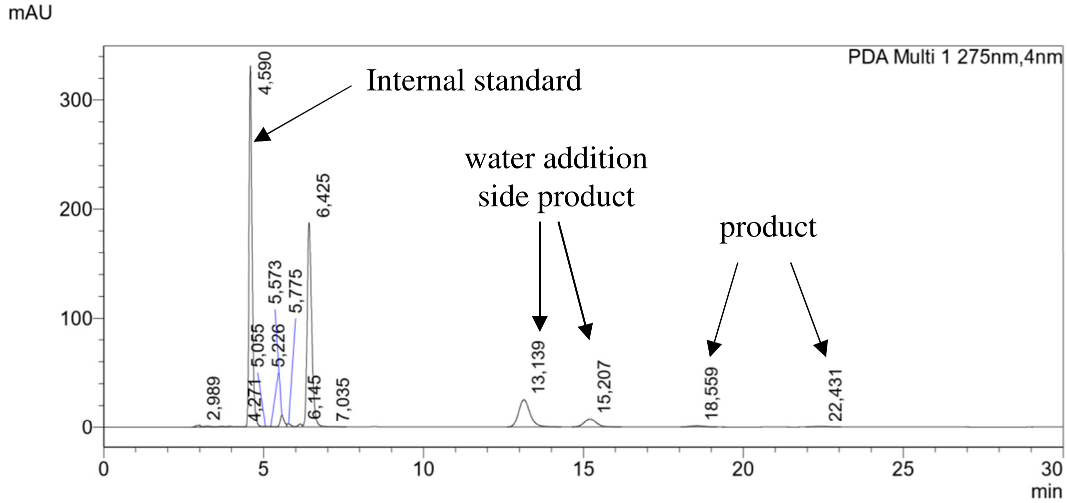


**S10.4. Catalyst: $Cu^{2+}$⊂CgmR**

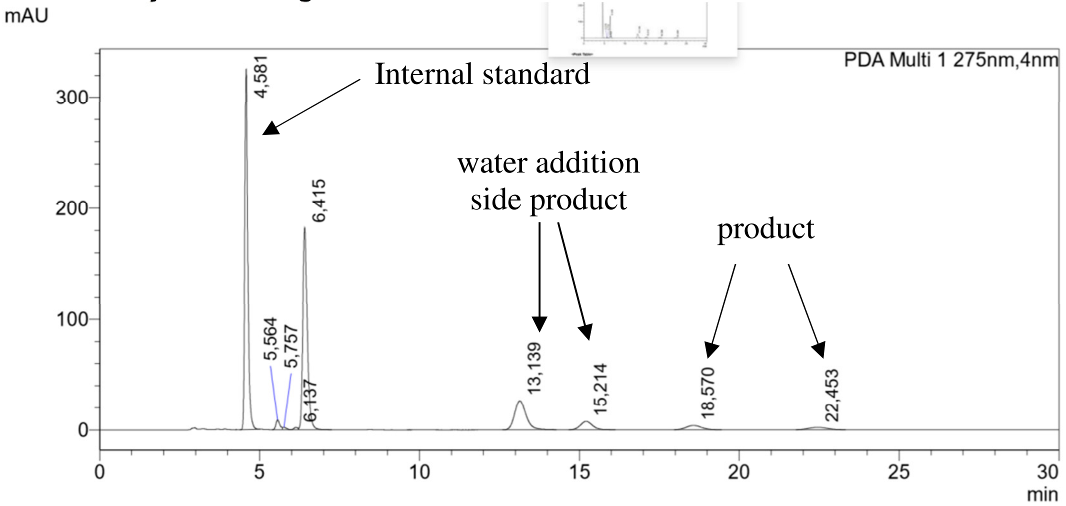


**S10.5. Catalyst: $Cu^{2+}$⊂QacR**

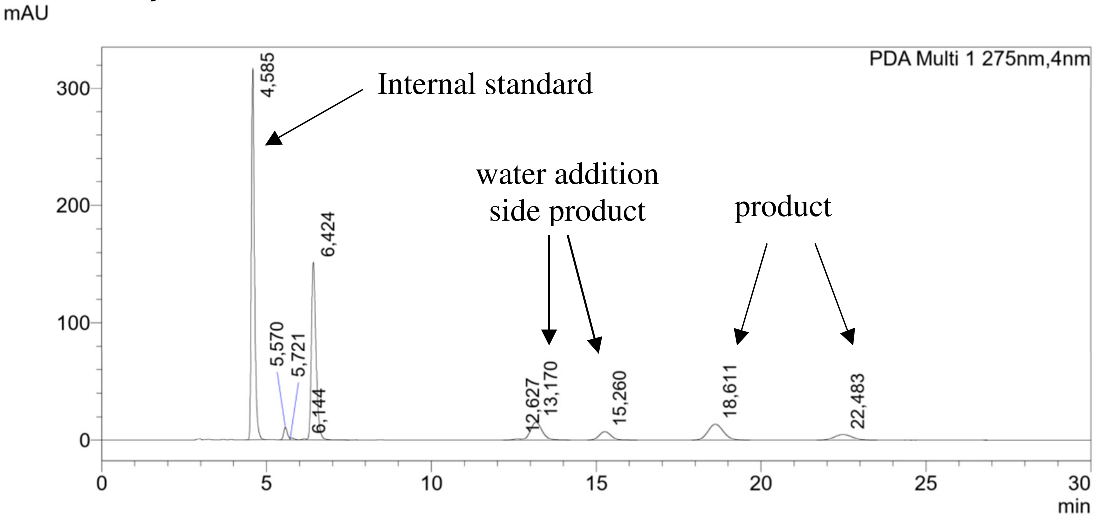

**Figure S11: calibration curve and chiral HPLC traces obtained for biocatalytic reactions with product 3f**

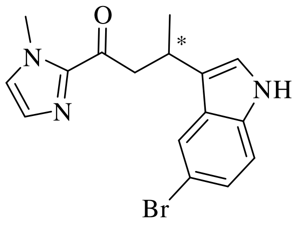

**3f**

**S11.1. Calibration curve**

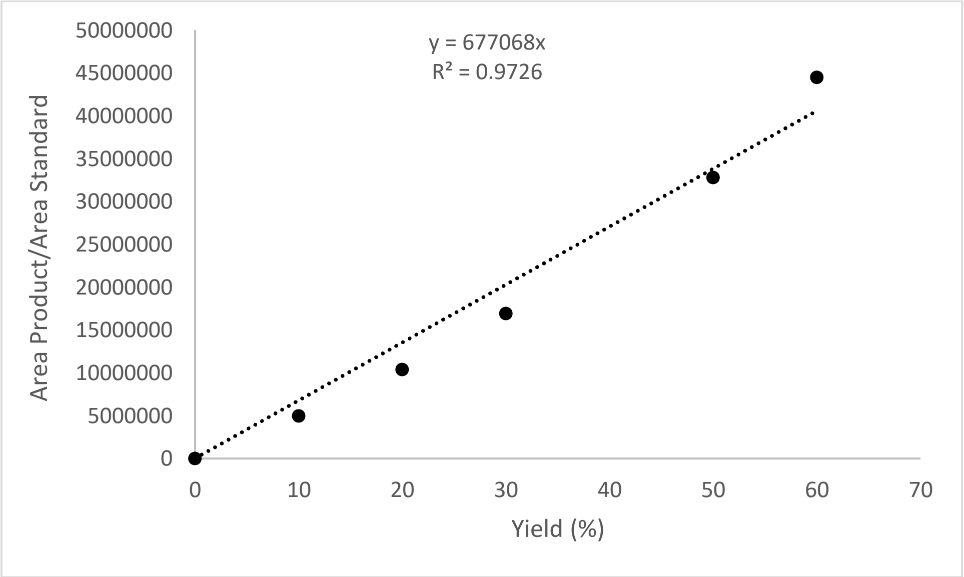


**S11.2. Catalyst: $Cu(NO_3)_2$**

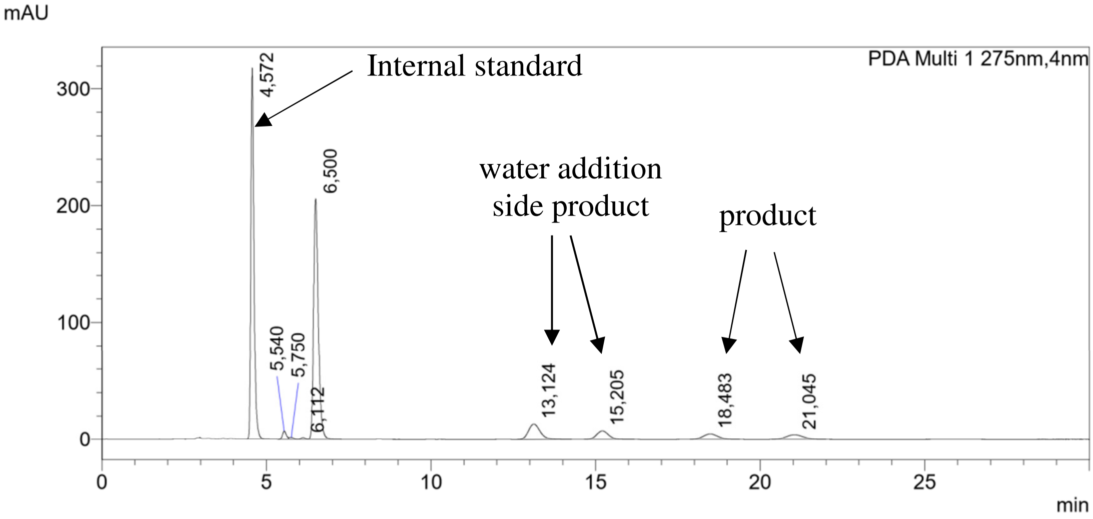

**S11.3. Catalyst: $Cu^{2+}$⊂RamR**

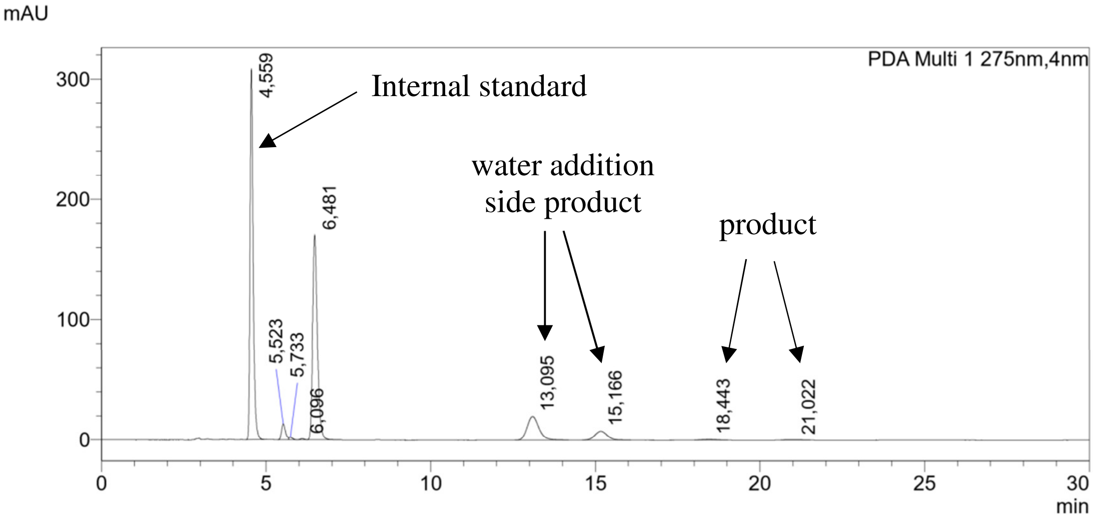


**S11.4. Catalyst: $Cu^{2+}$⊂CgmR**

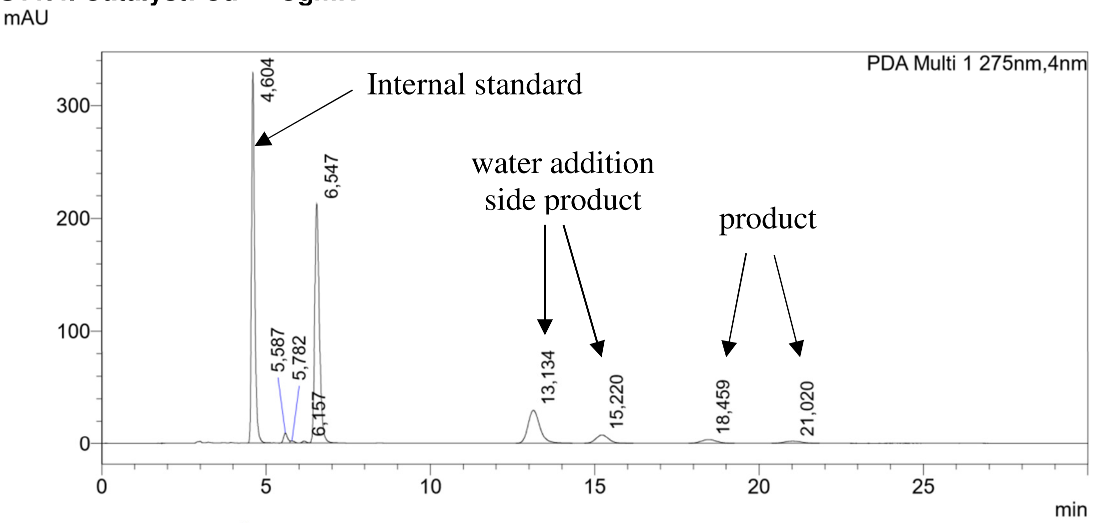


**S11.5. Catalyst: $Cu^{2+}$⊂QacR**

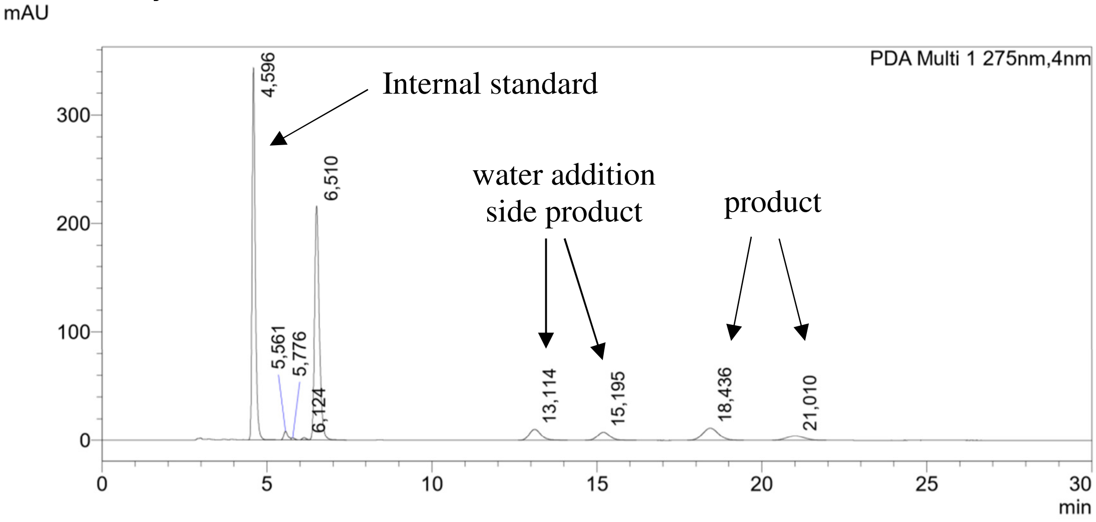

**Figure S12: calibration curve and chiral HPLC traces obtained for biocatalytic reactions with product 3g**

### S12.1. Calibration curve

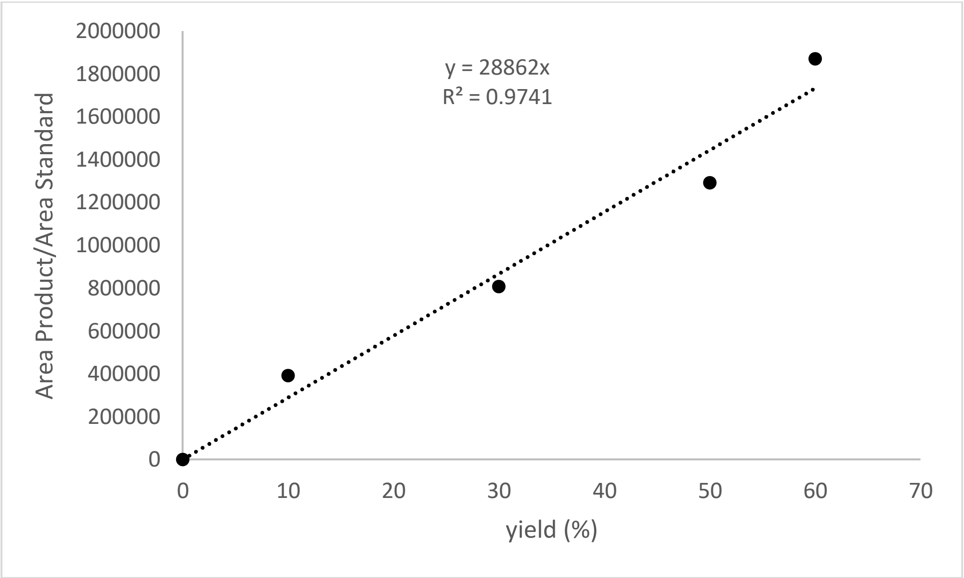


### S12.2. Catalyst: $Cu(NO_3)_2$

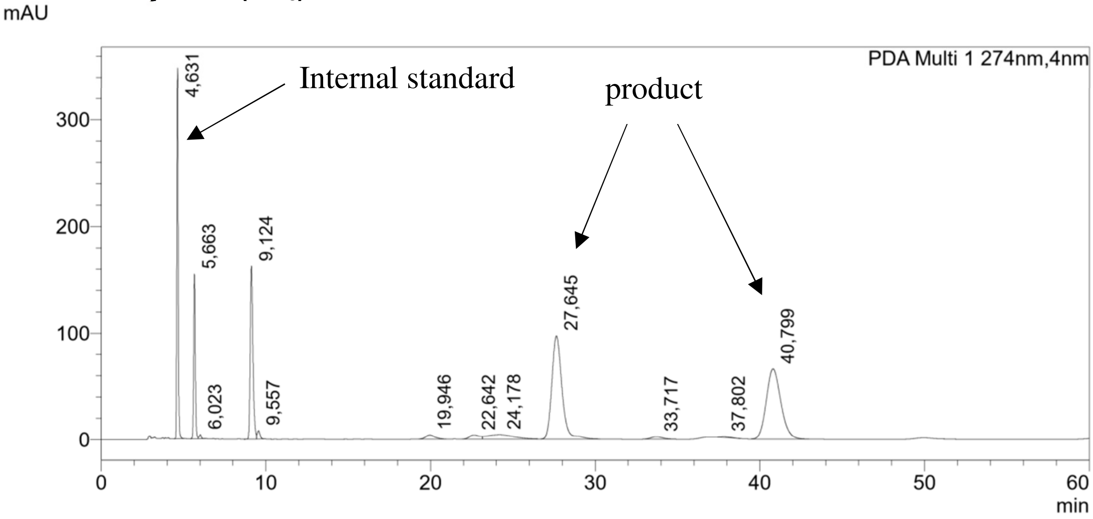

**S12.3. Catalyst: $Cu^{2+}$⊂RamR**

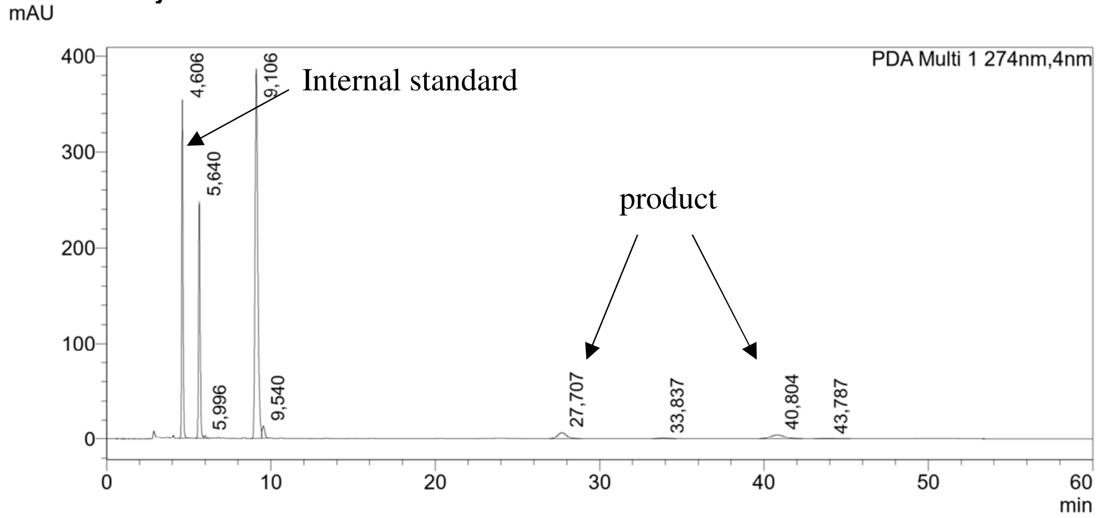


**S12.4. Catalyst: $Cu^{2+}$⊂CgmR**

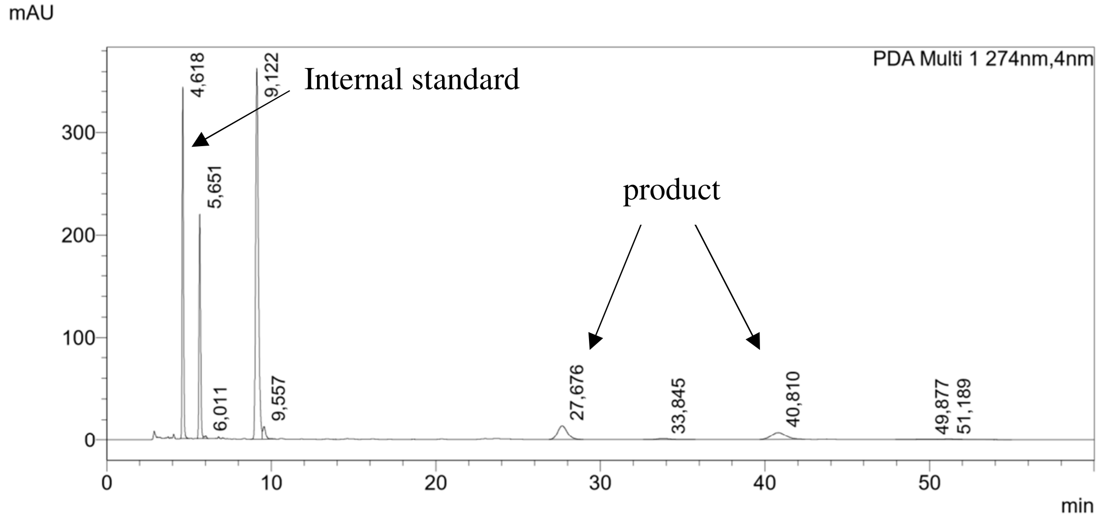


**S12.5. Catalyst: $Cu^{2+}$⊂QacR**

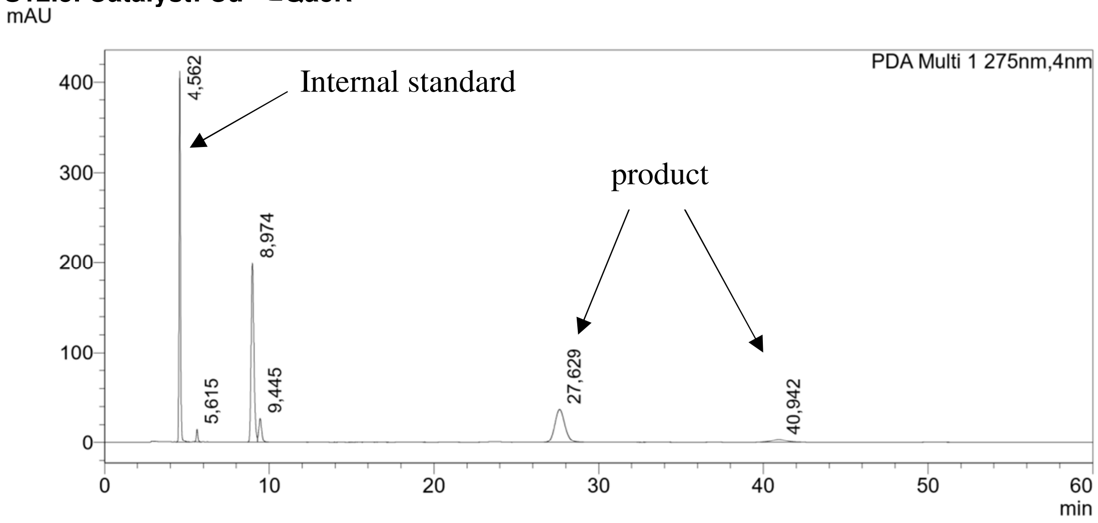

**Figure S13: calibration curve and chiral HPLC traces obtained for biocatalytic reactions with product 3h**

**S13.1. Calibration curve**

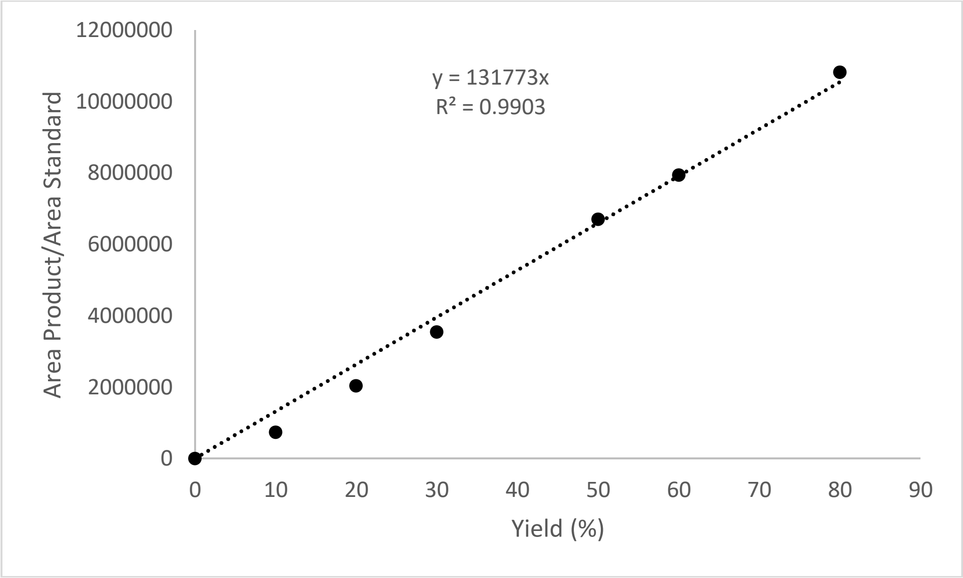


**S13.2. Catalyst: $Cu(NO_3)_2$**

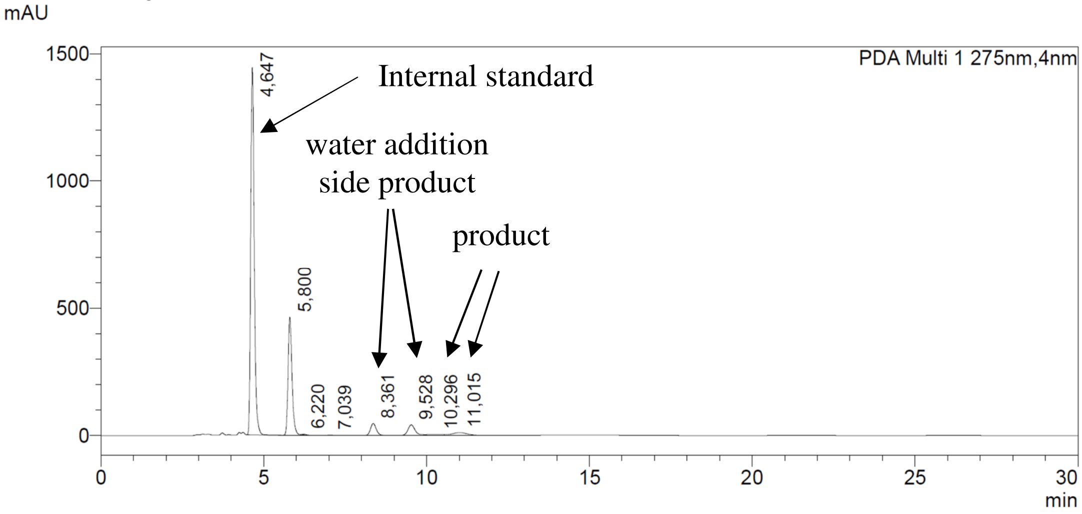

**S13.3. Catalyst: $Cu^{2+}$⊂RamR**

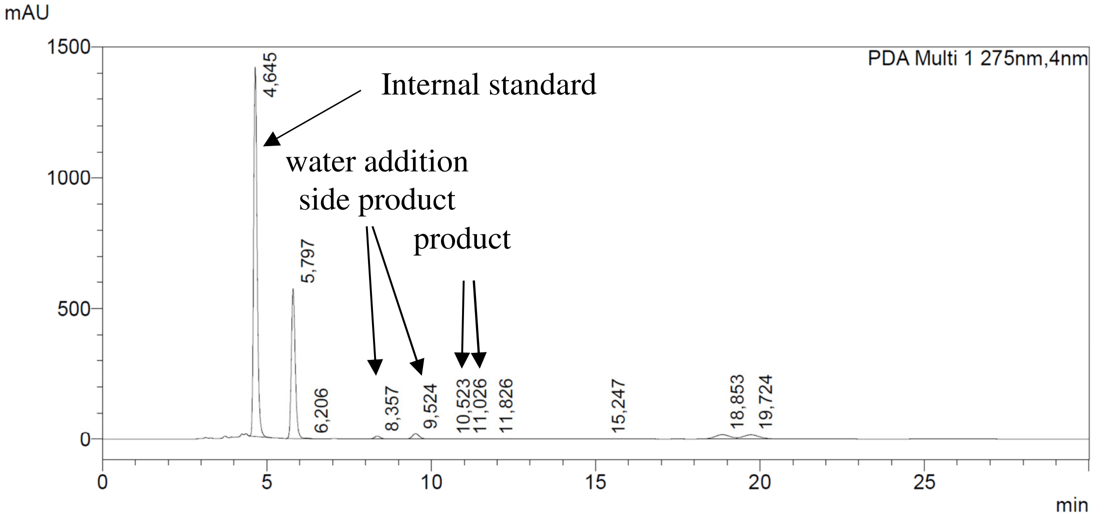


**S13.4. Catalyst: $Cu^{2+}$⊂CgmR**

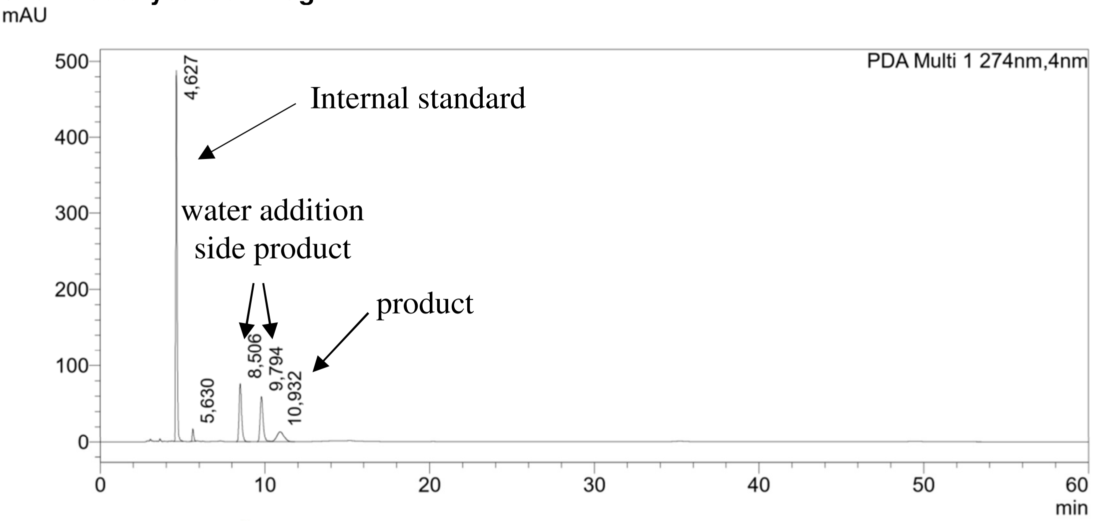


**S13.5. Catalyst: $Cu^{2+}$⊂QacR**

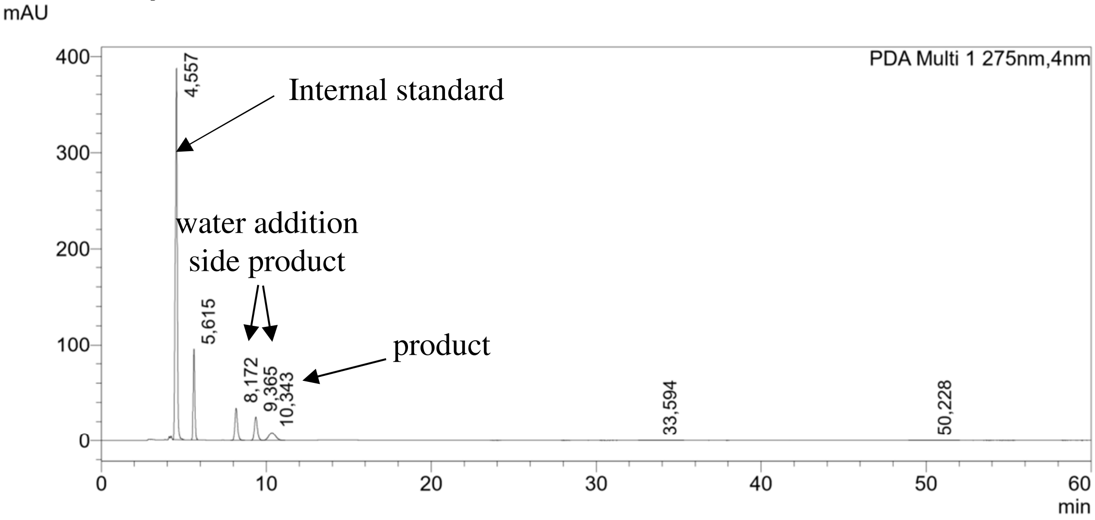

**Figure S14. Chiral HPLC traces for water addition product (4)**

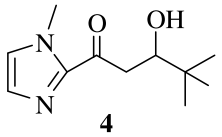

**4**

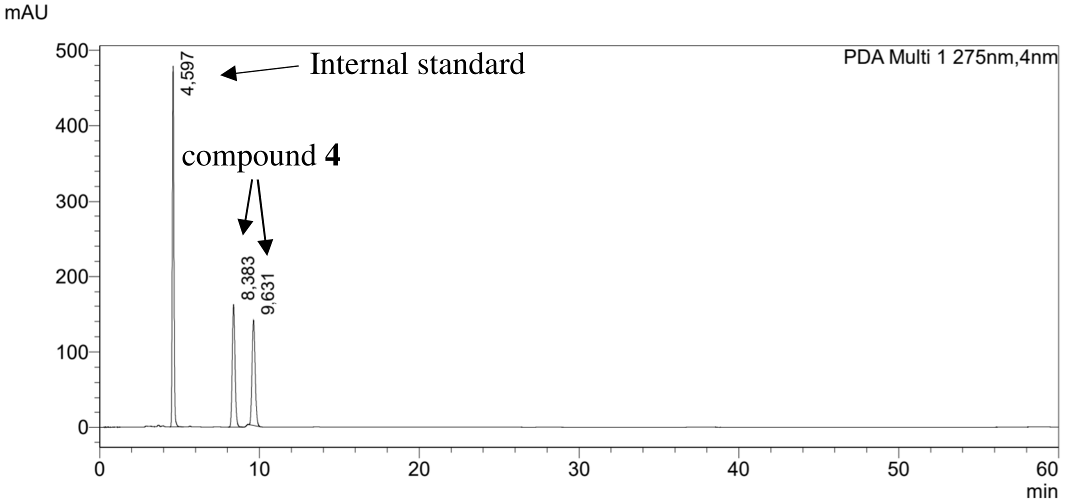


## 7. References

[1] V.P.A. and I.P. Beletskaya, *Top Organomet Chem 43 1–20.* **2009**, *48*, 11533–11542.

[2] J. Bos, W.R. Browne, A.J.M. Driessen, G. Roelfes, *J. Am. Chem. Soc.* **2015**, *137*, 9796–9799.

[3] D.A. Evans, K.R. Fandrick, H.J. Song, *J. Am. Chem. Soc.* **2005**, *127*, 8942-843.

[4] M. Bersellini, G. Roelfes, *Org. Biomol. Chem.* **2017**, *15*, 3069–3073.

[5] N. Duchemin, E. Benedetti, L. Bethge, S. Vonhoff, S. Klussmann, J.J. Vasseur, J. Cossy, M. Smietana, S. Arseniyadis, *Chem. Commun.* **2016,** *52,* 8604.

[6] A.J. Boersma, B.L. Feringa, G. Roelfes, *Angew. Chem. Int. Ed.* **2009**, *48*, 3346-3348.

[7] J.H. Yum, S. Park, R. Hiraga, I. Okamura, S. Notsu, H. Sugiyama, *Org. Biomol. Chem.* **2019**, *17*, 2548-2553.
